# The Caffeinated Brain Part 2: The Effect of Caffeine on Sleep-Related Electroencephalography (EEG)—A Systematic and Mechanistic Review

**DOI:** 10.3390/nu18081220

**Published:** 2026-04-13

**Authors:** James Chmiel, Donata Kurpas

**Affiliations:** 1 Faculty of Physical Culture and Health, Institute of Physical Culture Sciences, University of Szczecin, Al. Piastów 40B blok 6, 71-065 Szczecin, Poland; 2 Division of Research Methodology, Department of Nursing, Faculty of Nursing and Midwifery, Wroclaw Medical University, ul. Bartla 5, 51-618 Wrocław, Poland

**Keywords:** caffeine, electroencephalography, EEG, neurophysiology, nutrition, sleep

## Abstract

Introduction: Caffeine is the most widely consumed psychoactive stimulant worldwide and acts primarily through antagonism of adenosine A1 and A2A receptors, thereby reducing sleep pressure and promoting wakefulness. Although its alerting and performance-enhancing effects are well established, its influence on sleep-related electroencephalography (EEG) has been investigated across diverse paradigms with substantial methodological heterogeneity. This systematic and mechanistic review aimed to synthesize human evidence on how caffeine affects sleep architecture, quantitative sleep EEG, and neurophysiological markers of sleep homeostasis, and to interpret these findings within current models of adenosine-mediated sleep–wake regulation. Materials and Methods: A systematic search of PubMed/MEDLINE, Web of Science, Scopus, Embase, PsycINFO, ResearchGate, and Google Scholar was conducted for studies published between January 1980 and January 2026, with the final search performed on 10 January 2026. Eligible studies were original human investigations examining caffeine exposure or administration and reporting sleep-related EEG outcomes, including polysomnographic sleep staging, spectral EEG analyses, or other EEG-derived sleep metrics. Two reviewers independently screened records and assessed eligibility, with disagreements resolved by consensus. Data on study design, participant characteristics, caffeine interventions, EEG methodology, and outcomes were extracted using a predefined form. Risk of bias was evaluated using the RoB 2 and ROBINS-I tools. Owing to marked heterogeneity across studies, findings were synthesized narratively within a mechanistic interpretive framework. Results: Thirty-two studies were included. Across highly heterogeneous paradigms—including acute bedtime or evening dosing, daytime or repeated caffeine use before nocturnal sleep, administration during prolonged wakefulness followed by recovery sleep, withdrawal protocols, and ambulatory/home EEG monitoring—the most consistent finding was suppression of low-frequency NREM EEG activity, particularly slow-wave activity and the lowest delta frequencies. Caffeine frequently increased faster EEG activity, including sigma/spindle and beta ranges, producing a lighter, more aroused, and more wake-like sleep EEG profile. These effects were especially prominent during early-night NREM sleep and in recovery sleep after sleep deprivation, where caffeine attenuated the expected homeostatic rebound in low-frequency power. REM-related effects were less consistent, but some studies reported delayed REM timing and subtler alterations in REM EEG. Emerging evidence further suggests that caffeine increases EEG complexity and shifts sleep dynamics toward a more excitation-dominant state. Several studies indicated that quantitative EEG measures were more sensitive than conventional sleep-stage variables in detecting caffeine-related sleep disruption. Dose, timing, habitual caffeine use, withdrawal state, age, circadian context, and adenosinergic genetic variation, particularly involving ADORA2A, moderated the magnitude of effects. We also highlighted the connection between current results and sports and sports science. Conclusions: Caffeine reliably alters the neurophysiological architecture of human sleep in a direction consistent with reduced sleep depth and weakened homeostatic recovery. The overall evidence supports a mechanistic model centered on adenosine receptor antagonism, attenuation of sleep-pressure build-up and expression, and a shift toward greater cortical arousal during sleep. Sleep EEG appears to be a sensitive marker of these effects, often revealing physiological disruption even when conventional sleep architecture changes are modest. Future research should prioritize larger and more diverse samples, pharmacokinetic and pharmacogenetic characterization, and ecologically valid high-resolution sleep monitoring to clarify the real-world and functional consequences of caffeine-induced EEG changes.

## 1. Introduction

Caffeine is a naturally occurring psychoactive compound that belongs to the methylxanthine group and is widely consumed in coffee, tea, cocoa, and many commercial beverages. In neurobiological terms, caffeine acts mainly as a central nervous system stimulant because, at usual dietary doses, it blocks adenosine receptors, especially A1 and A2A receptors. Adenosine usually accumulates during wakefulness and contributes to sleep pressure, reduced arousal, and the subjective feeling of tiredness. By antagonizing this adenosine signaling, caffeine weakens one of the brain’s major sleep-promoting influences and therefore tends to increase wakefulness, mental activation, and fatigue resistance [1].

A large body of behavioral research shows that caffeine has a measurable effect on alertness, attention, and sleepiness. Human studies and reviews have consistently reported that caffeine reduces subjective sleepiness and improves behavioral performance, particularly on tasks that require sustained attention and vigilance. In one classic double-blind experiment, Zwyghuizen-Doorenbos and colleagues found that caffeine increased sleep latency on the Multiple Sleep Latency Test, which is an objective indicator of reduced daytime sleepiness, and improved auditory vigilance compared with placebo [2]. More recent work has shown similar effects under sleep-loss conditions: Quiquempoix et al. reported that caffeine alleviated sustained-attention deficits associated with total sleep deprivation, especially on psychomotor vigilance measures [3], while Killgore et al. found that repeated caffeine dosing helped maintain vigilance and attention during prolonged wakefulness, although it did not fully restore baseline performance [4]. Taken together, these studies show that caffeine does not merely make people feel more awake; it can produce observable behavioral improvements in alertness, resistance to sleepiness, and sustained attention, especially when normal arousal is reduced by fatigue or sleep deprivation.

Sleep electroencephalography, or sleep EEG, is one of the core scientific methods used to study sleep because it records the electrical activity of the brain from scalp electrodes throughout the sleep period. In both clinical and research settings, sleep EEG is usually part of polysomnography. This broader recording combines EEG with eye movements, muscle activity, respiration, and other physiological signals to identify sleep stages and sleep-related events. The EEG signal enables researchers to classify sleep into wakefulness, N1, N2, N3, and REM, as each stage has characteristic electrophysiological features. For example, N2 sleep is primarily defined by sleep spindles and K-complexes, whereas N3 sleep is characterized by dominant slow-wave or delta activity. In this way, sleep EEG provides the basic framework for describing sleep architecture across the night [5,6].

**Figure 1:**
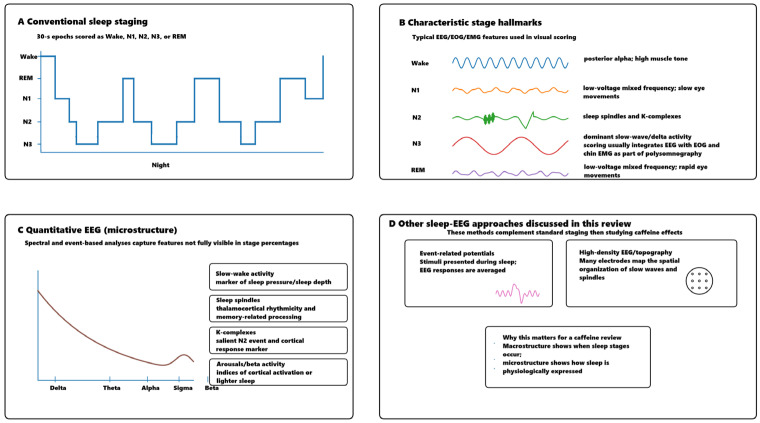
Sleep EEG approaches relevant to caffeine research.

There are several main types of sleep-related EEG studies. One necessary type is conventional sleep staging, also called macrostructural analysis, in which the night is divided into epochs, and each epoch is scored as wake, N1, N2, N3, or REM sleep according to standard criteria. This approach is used to quantify sleep onset, total sleep time, sleep efficiency, awakenings, and the proportion of each sleep stage. A second type is quantitative EEG, or spectral analysis, which examines the frequency composition and power of the EEG signal in bands such as delta, theta, alpha, sigma, and beta. This method is beneficial for studying sleep microstructure, including slow waves, spindle activity, and subtle changes in sleep depth that are not fully captured by standard staging. A third type is event-related potential research, in which sensory stimuli are presented during sleep, and the EEG response is averaged to study how the sleeping brain continues to process information in the absence of overt behavioral responses. Finally, more advanced approaches, such as high-density EEG, use many more scalp electrodes to map the spatial organization of slow waves and spindles and to investigate how sleep activity is distributed across cortical regions. Together, these methods allow sleep researchers to study both the overall architecture of sleep and the fine-grained neural dynamics within it [7,8,9]. Figure 1 shows different types of EEG staging.

From a sports-science perspective, this distinction between sleep macrostructure and sleep microstructure is especially important. In athletes and physically active individuals, recovery is not determined only by how long one sleeps or by the percentage of time spent in broad sleep stages, but also by the quality of the underlying neural oscillations that support restoration, plasticity, and next-day readiness. Quantitative sleep EEG is therefore highly relevant because slow-wave activity reflects the intensity of homeostatic sleep pressure and is considered a sensitive marker of biological recovery. In contrast, sleep spindles are linked to thalamocortical processing and sleep-dependent memory consolidation, including motor-skill consolidation. This matters in sport because training adaptation depends not only on acute performance capacity, but also on overnight recovery of the brain systems involved in fatigue regulation, learning, decision-making, and sensorimotor refinement. Consequently, if caffeine alters EEG microstructure even when conventional sleep staging shows only modest changes, sleep EEG may detect a recovery cost that standard sleep architecture measures could underestimate.

A clearer mechanistic link can also be made between adenosine receptor signaling and the EEG features used to characterize sleep. Adenosine is a central mediator of sleep homeostasis. As wakefulness is prolonged, adenosinergic signaling contributes to the increasing drive for sleep and to the cortical synchronization that is typically expressed during NREM sleep as prominent low-frequency activity, particularly slow-wave activity in the delta range. Because caffeine acts primarily as an antagonist at A1 and A2A receptors, it is biologically plausible that it modifies the expression of these oscillatory markers rather than affecting only visually scored sleep stages. In this context, quantitative EEG is especially informative, since it captures the spectral signatures through which sleep pressure, cortical arousal, and thalamocortical rhythm generation are expressed. Thus, examining EEG frequency bands provides a mechanistically relevant framework for understanding how caffeine may influence the neurophysiological quality of sleep.

If caffeine increases alertness and combats drowsiness, it must have widespread neurophysiological effects on the brain. Studies examining caffeine’s effects on sleep-related EEG are a valuable source of information on this effect. Over the last several decades, studies of caffeine and sleep-related EEG have accumulated across a wide range of paradigms, including acute bedtime challenges, daytime or evening dosing before nocturnal sleep, administration during prolonged wakefulness followed by recovery sleep, repeated daytime use and withdrawal protocols, and newer ambulatory or home-based EEG monitoring designs. This literature is methodologically rich but heterogeneous, making it challenging to identify the most consistent electrophysiological effects of caffeine and to interpret them mechanistically without a focused synthesis.

Accordingly, this review aims to systematically and mechanistically evaluate the effects of caffeine on sleep-related EEG in humans. Specifically, this review summarizes the characteristics of the included studies, the sleep-EEG paradigms and caffeine interventions employed, and the electrophysiological effects observed across nocturnal sleep, recovery sleep, and naturalistic monitoring contexts. It also considers whether the overall pattern of findings supports a coherent mechanistic account centered on adenosine antagonism, altered sleep homeostasis, circadian interaction, and inter-individual susceptibility.

## 2. Materials and Methods

### 2.1. Review Design

This review was conducted as a systematic and mechanistic review of the effects of caffeine on sleep-related electroencephalographic outcomes in humans. The aim was to identify, summarize, and interpret studies examining how caffeine influences sleep architecture, quantitative sleep EEG, and neurophysiological markers related to sleep homeostasis, cortical arousal, and recovery sleep. The review was designed and reported in accordance with the Preferred Reporting Items for Systematic Reviews and Meta-Analyses (PRISMA) guidelines.

### 2.2. Information Sources and Literature Search

A structured literature search was conducted across the following databases: PubMed/MEDLINE, Web of Science, Scopus, Embase, PsycINFO, ResearchGate, and Google Scholar. The final search was performed on 10 January 2026. Searches covered the period from January 1980 to January 2026.

The search strategy combined terms related to caffeine with terms related to sleep and electroencephalography. A representative search string was as follows:

(“caffeine” OR “caffeinated” OR “coffee”) AND (“sleep” OR “sleep architecture” OR “recovery sleep” OR “polysomnography”) AND (“EEG” OR “electroencephalography” OR “sleep EEG” OR “spectral analysis” OR “quantitative EEG” OR “slow-wave activity” OR “SWA” OR “spindle” OR “sigma activity”)

The search strategy was adapted as necessary for each database. In addition to database searching, backward and forward citation tracking were performed for all eligible articles and relevant reviews. Searches of similar articles, cited references, and citing records were also conducted to identify additional studies. Records were imported into EndNote for reference management and duplicate removal.

### 2.3. Eligibility Criteria

Studies were considered eligible if they met all of the following criteria:

1. They were original research studies in humans.
2. They examined caffeine exposure or administration as an experimental intervention or observational exposure;
3. They included sleep-related EEG outcomes, including conventional polysomnographic sleep staging, quantitative EEG or spectral analyses, or other EEG-derived sleep metrics obtained during nocturnal sleep, naps, recovery sleep, or ambulatory/home-based sleep recordings.
4. Only full-text articles published in English were eligible for inclusion.

Studies were excluded if they:

1. were conducted in animals;
2. did not report sleep-related EEG outcomes;
3. did not evaluate caffeine as a relevant intervention or exposure;
4. were reviews, editorials, letters, conference abstracts, protocols, or case reports.

### 2.4. Study Selection

After duplicate removal in EndNote, the remaining records underwent title and abstract screening, followed by full-text assessment of potentially relevant articles. Two independent reviewers selected studies according to predefined eligibility criteria. Disagreements were resolved through discussion and consensus with a third reviewer.

### 2.5. Data Extraction

Data were extracted from each included study using a predefined extraction form. Extracted variables included:

- study design and experimental paradigm;
- participant characteristics, including age, sex, health status, habitual caffeine use, and screening procedures;
- caffeine intervention characteristics, including dose, formulation, route or vehicle, timing relative to sleep, and whether dosing was acute, repeated, or part of a withdrawal design;
- sleep-recording methodology, including laboratory polysomnography, ambulatory EEG, or wearable/home-based systems;
- EEG and sleep outcomes, including sleep architecture variables, NREM and REM spectral power, slow-wave activity (SWA), sigma/spindle activity, beta activity, and any advanced signal-complexity or dynamical metrics;
- factors related to inter-individual variability, such as genotype, subjective caffeine sensitivity, age group, or trait sleep vulnerability.

Data extraction was performed by one reviewer and verified by a second reviewer. Any discrepancies were resolved by discussion with a third reviewer.

Information on sleep-staging methodology was also extracted when reported, including the scoring framework used (e.g., Rechtschaffen and Kales or AASM criteria), epoch length, and whether deep sleep was reported as stage 3, stage 4, slow-wave sleep (SWS), or N3. Because the included literature spans different historical periods and scoring conventions, these variables were considered when interpreting sleep-architecture outcomes, particularly for deep NREM sleep, where older studies often reported stage 3/4 or SWS rather than N3.

### 2.6. Quality Assessment/Risk of Bias

The methodological quality and risk of bias of the included studies were assessed using the ROB-2 and ROBINS-I tools.

### 2.7. Data Synthesis

Given the marked heterogeneity across studies in participant populations, caffeine regimens, EEG recording methods, and reported outcomes, a quantitative meta-analysis was not undertaken. Instead, findings were synthesized narratively.

The synthesis was organized around recurring domains identified across the literature: participant characteristics, sleep-related EEG paradigms, caffeine interventions, and caffeine’s effects on sleep-related EEG. Particular attention was given to mechanistically informative patterns, including impact on low-frequency NREM activity, recovery-sleep EEG after prolonged wakefulness, higher-frequency activity associated with cortical arousal, REM-related effects, and individual differences linked to habitual use, age, or adenosinergic biology.

### 2.8. Mechanistic Review Approach

In addition to systematically summarizing the human sleep-EEG literature, the review incorporated a mechanistic interpretive framework. Mechanistic interpretation focused on whether the observed EEG findings were consistent with current models of adenosine-mediated sleep homeostasis, receptor-level caffeine action, and downstream changes in neural arousal and sleep-depth regulation. This component was used to contextualize the physiological significance of the observed EEG patterns, particularly reductions in low-frequency activity and shifts toward faster, more wake-like sleep EEG dynamics.

### 2.9. Registration of the Review

This review has been registered in the PROSPERO database (CRD420261354163).

## 3. Results

**Figure 2:**
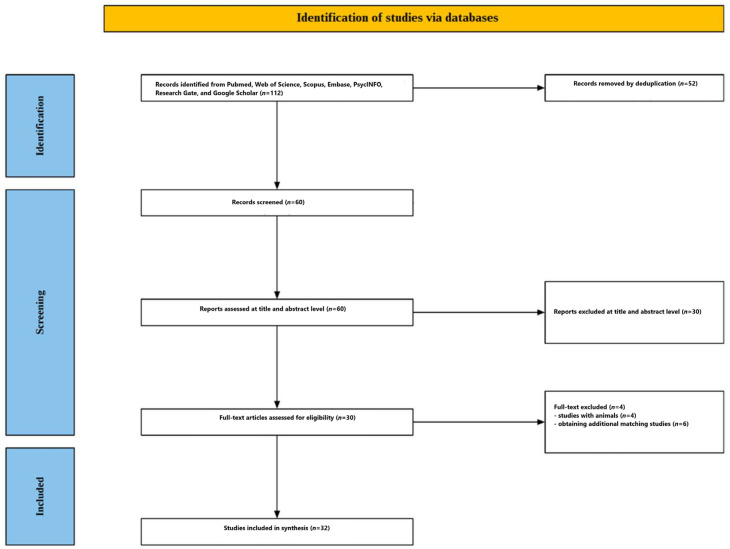
Flowchart depicting the phases of the systematic review.

**Figure 3:**
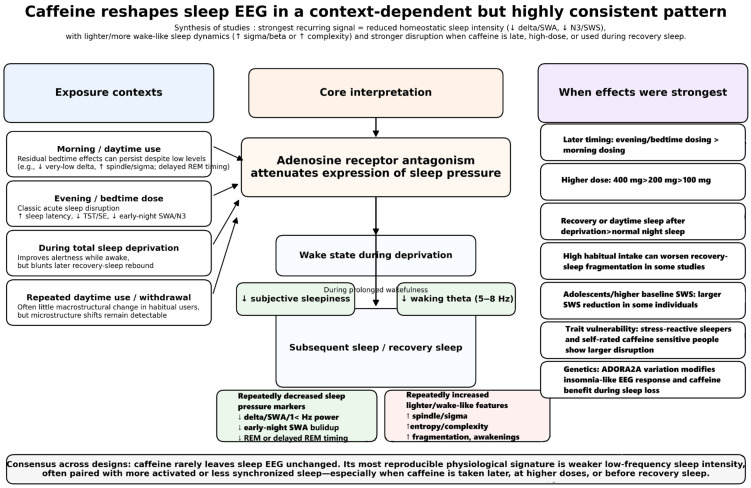
A graphical representation of the results.

Figure 2 summarizes the screening process. A search of research databases yielded 112 articles. After deduplication using EndNote, 60 articles remained for abstract and title evaluation. Thirty articles were excluded at this stage. The remaining 30 articles were submitted for full-text evaluation. Four studies were excluded at this stage because they tested caffeine effects in animals. Twenty-six studies were found that matched the review topic. Furthermore, a search for similar, cited, and citing articles yielded an additional six articles. Ultimately, 32 studies were included in this review [10,11,12,13,14,15,16,17,18,19,20,21,22,23,24,25,26,27,28,29,30,31,32,33,34,35,36,37,38,39,40,41]. The included studies are presented in Table 1, Table 2 and Table 3. A graphical representation of the results is provided in Figure 3.

### 3.1. Participant Characteristics

Across the included studies, participants were typically healthy individuals screened to exclude medical, psychiatric, neurologic, and sleep disorders, as well as factors known to alter sleep or caffeine metabolism, such as smoking, shift work, recent transmeridian travel, psychoactive medication use, and, in some studies, extreme chronotype or high alcohol intake [10,17,22,23,27,33,39,40]. Most laboratory studies also required regular sleep schedules before testing, verified with sleep diaries and/or actigraphy, and several imposed caffeine abstinence periods to minimize tolerance or withdrawal confounding [10,13,14,15,25,30,36].

A prominent feature of the literature is the predominance of young healthy adults, especially men. Several classic PSG/EEG studies enrolled only young male participants, often university-aged, with mean ages in the early-to-mid 20s and modest habitual caffeine use [10,13,14,15,24,26,28,33,35,38]. Sample sizes in these tightly controlled mechanistic studies were often small, ranging from 8 to 22 participants [10,13,14,15,19,20,24,26], whereas more recent studies tended to include larger samples and both sexes, for example 40 adults aged 20–58 years in a complexity/criticality EEG study [11], 41 adults aged 18–55 years in a total sleep deprivation recovery study [12], and 47 adults in a dose-by-age experiment [23]. Despite this broader inclusion in newer work, male-only designs remained common, particularly in studies seeking to reduce hormonal variability or maximize experimental control [13,24,25,27,33,35,40].

Habitual caffeine exposure was usually low to moderate rather than extreme. Many studies recruited moderate consumers, often operationalized as roughly 1–3 caffeinated beverages per day or below ~300–600 mg/day, while excluding very high users to reduce tolerance and withdrawal effects [10,11,14,15,17,18,22,23,27,33,35,40]. Some protocols specifically targeted low or modest users [10,13,24,38], whereas others examined variation in habitual intake as a moderator of sleep disruption [12,16,20]. A few studies deliberately enrolled habitual daily users to model real-world daytime caffeine use and withdrawal [27,33,35]. In contrast, others included moderate-use samples but required short-term or multi-day abstinence before laboratory testing [10,13,25,30,36].

Although young adults dominated the evidence base, the literature also included adolescents, middle-aged adults, and older adults. Adolescent samples were examined in both naturalistic and laboratory settings, including 98 adolescents aged 11–17 years in a home EEG diary study and 18 male adolescents aged 14–17 years in a placebo-controlled crossover experiment [21,25]. Multiple studies explicitly compared younger and middle-aged adults, typically pairing participants in their 20s with those in their 40s to late 50s to test whether age modified caffeine’s effects on sleep EEG [11,17,22,23]. Older adults were much less frequently represented, but one early PSG study focused specifically on six habitual caffeine users aged 50–63 years and suggested greater sensitivity to bedtime caffeine in the older subgroup [19].

Sex representation varied substantially by study. Many early experiments were restricted to men [10,13,14,15,19,20,24,25,26,27,28,33,35,38,40,41], but several later studies included both women and men in relatively balanced proportions [11,12,17,18,22,23,29,37,39]. When women were included, some studies further controlled for menstrual phase, hormonal contraception, menopausal status, or hormone replacement therapy to reduce endocrine confounding [17,18,22,23].

The participant profile across this literature is best characterized as healthy, non-smoking, regular-sleeping, low-to-moderate caffeine-consuming volunteers, with the strongest representation coming from young adult males studied under tightly controlled laboratory conditions [10,13,14,15,24,26,28,33]. This improves internal validity for detecting caffeine-related EEG effects, but it also means that generalizability to women, heavy caffeine users, clinical populations, and older adults remains comparatively limited [19,21,22,23,39,40].

Taken together, the available evidence supports only a limited form of population-level inference. The best-represented subgroup across this literature is healthy young adult men with regular sleep schedules and low-to-moderate habitual caffeine intake. By contrast, women, older adults, adolescents, heavy caffeine users, and individuals with clinical sleep disorders were included only sporadically and usually in small samples. For this reason, the current review cannot treat subgroup-specific conclusions as equally well established across populations. Rather, the strongest conclusion is that caffeine reliably modifies sleep EEG in healthy adults under controlled conditions. In contrast, the magnitude, timing dependence, and functional significance of these effects in underrepresented groups remain less certain. Because subgroup representation was sparse and methodologically heterogeneous, a formal subgroup analysis was not feasible; however, the available studies do suggest that age, habitual intake, trait sensitivity, circadian context, and adenosinergic genotype may moderate the EEG response to caffeine and should be considered when interpreting external validity.

### 3.2. Sleep-Related EEG Paradigms

The sleep-related EEG paradigms used across the included studies were diverse, Still, they can be grouped into a few recurring experimental approaches: (1) nocturnal sleep after acute caffeine administration, (2) recovery sleep after total sleep deprivation, (3) daytime recovery sleep under strong circadian wake promotion, (4) nap- and MSLT-based paradigms, and (5) repeated daytime caffeine or withdrawal protocols designed to probe adaptation and circadian effects [10,11,12,13,14,15,17,18,27,29,33,35].

A large portion of the literature used standard overnight nocturnal polysomnography following acute caffeine administration before habitual bedtime. In these paradigms, caffeine was typically given in the evening or at bedtime, and sleep was then recorded during an 8 h nocturnal sleep opportunity using conventional EEG/EOG/EMG-based PSG. This approach was used to examine how caffeine alters normal sleep architecture and NREM/REM spectral composition under relatively typical sleep conditions [13,14,19,20,22,23,26,37,39,41]. Some studies administered caffeine immediately before lights-out [14,19,26,41]. In contrast, others used split evening doses, often 100 mg given 3 h and 1 h before bedtime, to mimic realistic pre-sleep consumption and to sustain drug exposure into the sleep episode [17,18,22,23]. One study specifically tested morning caffeine and then recorded sleep at the next habitual bedtime, thereby assessing whether low residual caffeine levels were still sufficient to alter nocturnal EEG dynamics [15]. Another study used a delayed pulsatile-release capsule so that plasma caffeine rose during the sleep episode itself, allowing simultaneous modeling of concentration–effect relationships for EEG delta power and cardiac autonomic activity during a 4 h sleep opportunity [13].

A second major paradigm involved recovery sleep after prolonged wakefulness or total sleep deprivation (TSD). These protocols were especially important for testing caffeine’s effects on sleep homeostasis. Participants typically underwent 27–40 h, and in one case 64 h, of continuous wakefulness under close supervision, after which recovery sleep was recorded with PSG and spectral EEG analysis [10,12,16,24,30,34,36]. In these studies, caffeine was usually administered during the deprivation period rather than immediately before sleep, allowing the question to be asked whether caffeine merely promotes wakefulness or also reduces the subsequent homeostatic rebound in slow-wave activity and low-frequency nonREM power [10,30,34,36]. These paradigms often combined repeated waking EEG during sleep deprivation with recovery-night PSG, thereby linking the build-up of waking theta activity to subsequent changes in nonREM delta or <1 Hz power during recovery sleep [10,30,36]. Recovery nights were typically extended (e.g., 10.5 h) to capture the full rebound response [10,30,36], although some studies used more standard 8 h or ad libitum recovery windows [12,16,24].

A related but distinct paradigm examined daytime recovery sleep after one night of sleep deprivation. Here, sleep occurred during a circadian phase that naturally promotes wakefulness, making the model especially sensitive to sleep-disrupting effects of caffeine [17,18]. In these studies, participants first remained awake all night. Then they attempted a daytime recovery sleep episode approximately 25 h after waking, with caffeine administered in two 100 mg doses before daytime bedtime [17,18]. PSG and quantitative NREM EEG were then used to evaluate whether caffeine impaired sleep more strongly in this circadian context than during habitual night sleep. This design was particularly useful for separating homeostatic pressure from circadian timing effects, and it showed that caffeine could markedly worsen sleep consolidation and reduce low-frequency synchronization during daytime recovery sleep [17,18].

Several studies used nap-based EEG paradigms rather than full nocturnal sleep. Some focused on prophylactic naps or short sleep opportunities before or during extended wakefulness, combining EEG-defined sleep with subsequent vigilance testing [28,29,32]. In one experiment, participants received caffeine before a fixed-duration nocturnal nap, allowing sleep time to be held constant while examining whether caffeine-induced changes in sleep stage composition and metabolic activation reduced the nap’s later restorative value [28]. In another, repeated 2 h naps were scheduled every 12 h during 88 h of extended wakefulness, and PSG was used to determine whether low-dose hourly caffeine prevented sleep inertia immediately after abrupt awakenings [29]. Related operational studies used EEG-defined Multiple Sleep Latency Tests (MSLTs) as objective markers of sleep propensity, often alongside nocturnal PSG, to evaluate nap latency or alertness during continuous operations with or without caffeine [32,41]. In these paradigms, the EEG outcome was less often spectral power and more often latency to stage 1 or stage 2 sleep, though sleep-stage architecture was also scored when full nap recordings were available [29,32].

Another important group of studies used repeated daytime caffeine administration over several days to model habitual use, tolerance, and withdrawal. In these protocols, participants took caffeine multiple times per day, usually in the morning, midday, and afternoon, for 8–10 days, and then underwent PSG and spectral EEG either during continued caffeine intake or after abrupt cessation [27,33,35]. These studies were designed to reflect real-world caffeine consumption rather than an isolated laboratory challenge. Their sleep recordings were often embedded within highly controlled circadian laboratory routines, including dim light, fixed posture, controlled meals, and removal of time cues [27,33,35]. One study used an evening nap at a circadian wake-promoting time to probe sleep pressure during withdrawal [27], while another scheduled the main sleep episode near the circadian peak of REM promotion, approximately 5 h after habitual bedtime, to determine whether regular daytime caffeine altered REM timing rather than gross sleep architecture [35]. These paradigms broadened the literature beyond acute caffeine at bedtime. They showed that microstructural EEG measures, such as sigma activity or REM latency, may be more sensitive than conventional sleep-stage scoring under habitual-use conditions [33,35].

The recording technology also varied meaningfully across studies. Most mechanistic work relied on full laboratory PSG with multi-channel EEG, EOG, and EMG, often using 10–20 system derivations and artifact-controlled FFT-based spectral analyses focused on NREM sleep [10,13,14,15,17,18,22,23,30,34,36]. Many studies centered analyses on the C3-A2 derivation for comparability and because it was standard in earlier spectral work [10,13,15,30,34]. Others examined broader topographic distributions across frontal, central, parietal, occipital, or prefrontal regions to identify spatial specificity in caffeine effects [14,17,22,36]. More recent studies expanded beyond canonical power spectra to include aperiodic-adjusted spectra, entropy/complexity measures, DFA scaling, and machine-learning classification of NREM versus REM caffeine effects [11]. In contrast, ecologically oriented or field-based studies used reduced-channel devices, such as a Dreem headband [12], a single-channel home EEG system in adolescents [21], or a wireless forehead monitor for at-home caffeine timing experiments [37]. These approaches enabled larger or more naturalistic samples, though often with less detailed topographic information than full PSG.

Across paradigms, the principal EEG outcomes were usually spectral markers of sleep depth and cortical activation. Most studies emphasized NREM low-frequency power, especially slow-wave activity (delta power), as the primary index of sleep homeostasis [10,13,14,30,34]. Several also examined sigma/spindle activities, theta/alpha ranges, beta activity, or very slow <1 Hz power to characterize caffeine-related shifts toward lighter or more wake-like sleep [10,14,15,17,22,23,36,40]. REM sleep was less frequently the primary target, but some paradigms specifically assessed REM latency, REM spectral changes, or REM vulnerability under circadian manipulation [11,17,18,35]. Thus, while the paradigms differed in timing, dose scheduling, and recording context, most converged on a shared framework: caffeine’s effects on sleep-related EEG were examined by challenging either normal nocturnal sleep, homeostatic rebound sleep, or circadian-misaligned sleep, and then quantifying how EEG signatures of sleep intensity, synchronization, and stage organization were altered [10,11,12,13,14,15,16,17,18,23,25].

### 3.3. Caffeine Interventions

The caffeine interventions used across the included studies varied substantially in dose, timing, route of administration, and experimental purpose, yet several broad patterns emerged. Most studies tested acute caffeine exposure in placebo-controlled crossover designs, typically comparing one caffeine condition with a matched placebo condition [10,11,12,13,14,15,17,18,19,20,22,23,25,27,29,30,33,34,35,36,37,39,40]. A smaller number used multi-dose or dose-response designs to determine whether sleep and EEG effects scaled with the amount of caffeine administered [16,19,23,26,37,40,41], while others examined repeated daytime caffeine use or withdrawal to model habitual consumption more realistically [27,33,35,38].

A common intervention strategy was the administration of moderate acute doses, most often 100–200 mg, around the time of sleep. Several studies used 200 mg total as the principal manipulation, either as a single dose or divided into two 100 mg capsules [11,15,17,18,22]. In evening or bedtime sleep paradigms, the dose was frequently split into 100 mg 3 h before bedtime and 100 mg 1 h before bedtime, a schedule intended to mimic realistic evening intake while maintaining appreciable caffeine levels during sleep [17,18,22,23]. Other studies used 100 mg at bedtime to test whether even a relatively low dose could modify sleep architecture and EEG slow-wave activity [14]. In adolescents, the experimental dose was reduced to 80 mg, given 4 h before bedtime, to approximate the amount in a small energy drink or a typical adolescent intake [25].

Several studies instead used larger acute doses to maximize sleep disruption or establish dose-response effects. Bedtime doses of 300 mg [19], 400 mg [27], or 4–8 mg/kg delivered as coffee [41] produced more pronounced impairment of sleep initiation and consolidation. In a classic coffee study, caffeine exposure ranged from approximately 1.1, 2.3, and 4.6 mg/kg through 1-, 2-, and 4-cup equivalents, with an additional pure-caffeine condition to isolate caffeine from other coffee constituents [26]. A more recent home-based trial also explicitly contrasted a typical dose (100 mg) with a high dose (400 mg) administered 12, 8, or 4 h before bedtime, showing that only the higher dose reliably disrupted sleep [40]. Together, these studies indicate that caffeine interventions were often designed either to reflect ecologically plausible consumption levels or to create a robust insomnia-like challenge.

In sleep-deprivation paradigms, caffeine was generally administered during sustained wakefulness rather than immediately before recovery sleep. This intervention logic was central to studies of sleep homeostasis because it allowed investigators to test whether caffeine reduced the accumulation of sleep pressure and altered the subsequent recovery-sleep EEG rebound [10,12,16,24,30,34,36]. A widely used protocol involved two 200 mg doses, given after 11 and 23 h of wakefulness [10,30,34,36]. Another total sleep deprivation study used 2.5 mg/kg caffeine administered in a decaffeinated beverage at 08:30 and 14:30, repeated across the deprivation protocol, with the final dose given 6.5 h before recovery bedtime to model a realistic operational scenario [12]. Operational and military studies tended to use repeated overnight dosing, including chewing gum delivering effective total doses of about 255 mg, 510 mg, or 765 mg across three administrations [16], or 300 mg of slow-release caffeine twice daily during prolonged wakefulness [24]. These interventions were not primarily intended to test bedtime caffeine per se, but rather to determine whether caffeine used to maintain alertness during deprivation would carry forward into altered recovery sleep EEG.

The literature also included interventions designed to model habitual daytime caffeine use. In these studies, participants typically consumed caffeine three times daily, often at 150 mg per dose, for a total of 450 mg/day, over 8–10 days [27,33,35]. Capsules were usually taken at standardized times after awakening—for example, approximately 45, 255, and 475 min after wake-up—to represent morning, midday, and afternoon use while avoiding evening dosing [27,33,35]. This approach allowed researchers to examine whether repeated daytime intake changed nocturnal PSG and EEG even when the last dose occurred many hours before sleep, and whether abrupt cessation induced detectable withdrawal-related changes [27,33,35]. One related insomnia-model study used 400 mg sustained-release caffeine three times daily, including a bedtime dose, across multiple days to induce repeated hyperarousal and chronic sleep disruption [38].

Timing of the intervention relative to sleep was one of the most important design dimensions. Some studies delivered caffeine immediately before lights-out [14,19,26,41], maximizing exposure during sleep onset and the first NREM cycle. Others gave caffeine 1–6 h before bedtime to examine how late intake could still disrupt sleep [22,23,25,37,40]. Morning-only dosing was also tested: one study administered 200 mg about 10 min after waking. Then it assessed sleep at the usual bedtime, showing that even low residual evening caffeine concentrations could influence the nocturnal EEG [15]. In another mechanistic design, researchers used a delayed pulsatile-release capsule containing 160 mg of caffeine, administered at habitual bedtime but engineered to release caffeine later, so that blood concentrations rose during the sleep opportunity itself [13]. This formulation was especially useful for linking real-time plasma caffeine concentration to EEG delta suppression and cardiac autonomic effects during sleep [13].

The route and formulation of caffeine also varied. Most laboratory studies used capsules, often containing caffeine anhydrous and matched with mannitol or lactose placebo [14,15,17,18,22,23,25,27,33,35,40]. Capsules were preferred because they support blinding and precise dosing. However, some studies used coffee-based administration [26,41], decaffeinated beverages spiked with caffeine [12], chewing gum [16], or hourly pills in prolonged wakefulness paradigms [29]. Slow-release formulations were also employed to sustain wake-promoting effects during extended operations [24,32,38]. These alternative formulations matter methodologically because they likely yield different pharmacokinetic profiles, which, in turn, can shape sleep timing and EEG effects.

Several studies explicitly incorporated pharmacokinetic verification into the intervention design. Salivary or plasma caffeine concentrations were measured in many protocols to confirm compliance, characterize absorption and clearance, and determine whether sleep effects occurred at residual or rising caffeine levels [10,13,14,15,22,23,25,27,30,33,35,40]. This was particularly important in studies arguing that caffeine altered sleep even at low concentrations at bedtime, such as the morning-dose design [15], or that effects persisted into sleep because measurable residual levels persisted after repeated daytime use [33,35]. In concentration–response work, measured plasma levels were themselves the main intervention variable of interest, allowing threshold estimation for EEG and autonomic outcomes [13].

Where reported, actual caffeine concentrations at or near the sleep period varied substantially across paradigms, helping contextualize the electrophysiological findings. In the morning-dose study, salivary caffeine at habitual bedtime averaged 2.7 ± 0.7 μmol/L, although individual values ranged up to 6.5 μmol/L, indicating that low but measurable residual levels could still be present at night [15]. In a delayed-release bedtime protocol specifically designed to raise caffeine during sleep, mean plasma caffeine was 4.19 ± 0.82 μmol/L at lights-off and increased to 8.00 ± 0.86 μmol/L by lights-on, with EEG delta suppression emerging at approximately 7.3–7.4 μmol/L [13]. After a 100 mg bedtime dose, salivary caffeine peaked at about 7.5 μmol/L roughly 1 h after administration and remained around 3.5 μmol/L by 06:00 [14]. In contrast, in recovery-sleep studies after prolonged wakefulness, concentrations could be very low by bedtime despite earlier dosing; for example, salivary caffeine fell to about 1.8 μmol/L 1 h before recovery sleep in one protocol [10]. In repeated daytime-use designs, measurable residual salivary caffeine remained detectable before sleep and on awakening, supporting the interpretation that nocturnal EEG changes may occur even when the final dose was taken many hours earlier [33,35].

Another notable feature was the frequent use of placebo-controlled crossover designs, in which each participant received both caffeine and placebo conditions [10,11,13,17,18,22,23,25,27,29,30,33,35,40,41]. This design minimized inter-individual variability in habitual sleep traits and caffeine sensitivity. In contrast, a smaller number of studies used between-group allocation for certain conditions, especially in operational or field paradigms [29,32], or divided participants by habitual caffeine use, age, genotype, or stress-vulnerability status to examine moderation of the caffeine effect [16,17,22,23,30,34,39]. Thus, the caffeine intervention was often not only the experimental manipulation but also a tool for probing trait vulnerability to sleep disruption.

### 3.4. The Effects of Caffeine on Sleep-Related EEG

The included studies show that caffeine alters sleep-related EEG in a systematic, yet context-dependent, manner. The most robust and reproducible finding is a reduction in low-frequency non-REM (NREM) activity, especially in the delta/slow-wave range, consistent with a reduction in sleep intensity and an attenuation of homeostatic sleep pressure [10,13,14,17,22,23,30,34]. Beyond this core effect, caffeine often also increases spindle-, sigma-, beta-, or other faster-frequency activity, suggesting a shift toward lighter and more wake-like cortical dynamics [10,14,15,17,22,23,30]. More recent work further indicates that caffeine can increase EEG complexity and entropy, particularly during NREM sleep, again consistent with reduced synchronization and a more activated brain state during sleep [11]. However, the precise pattern depends on the experimental paradigm, including whether sleep occurs at night or during daytime recovery, whether caffeine is given acutely or repeatedly, and whether sleep follows prolonged wakefulness.

#### 3.4.1. Suppression of Slow-Wave Activity and Low-Frequency NREM Power

The clearest finding across studies is that caffeine suppresses slow-wave activity (SWA) and related low-frequency EEG power during NREM sleep. This was observed in early nocturnal PSG studies using bedtime caffeine, where even 100 mg reduced SWA (0.75–4.5 Hz), especially in the first NREM episode, and slowed the normal buildup of SWA after sleep onset [14]. The effect was not a uniform suppression of all low frequencies. Still, it was strongest in the lowest delta frequencies, supporting the idea that caffeine selectively weakens the deepest and most homeostatically regulated aspects of sleep [14]. A similar pattern emerged after morning caffeine, with low residual evening levels still associated with reduced very-low-frequency NREM power at the subsequent habitual bedtime [15].

These nocturnal findings were strengthened by studies that directly linked caffeine concentration to sleep EEG. In a delayed-release protocol, caffeine significantly reduced NREM delta power in the 0.75–2.5 Hz range, and the suppression correlated negatively with plasma caffeine concentration, with a modeled threshold around 7.3–7.4 μmol/L above which delta suppression became reliable [13]. This study is important because it showed that caffeine-related EEG suppression is not merely categorical (caffeine vs placebo), but dose- and concentration-dependent during sleep itself [13].

The same general effect also appeared in studies comparing young and middle-aged adults. Across age groups, evening caffeine reduced low-frequency NREM power in frontal, central, and parietal derivations, with broadly similar effects in young and middle-aged adults on the main EEG measures [22]. In another age-by-dose study, caffeine reduced very low-frequency power and increased faster activity in both age groups. However, the age-by-dose interaction was clearer for conventional PSG sleep measures than for spectral EEG itself [23]. Thus, while aging modifies baseline sleep intensity, the basic caffeine-related suppression of low-frequency NREM activity seems relatively robust across adult age groups [22,23].

#### 3.4.2. Attenuation of the Homeostatic Rebound After Sleep Deprivation

The strongest support for a homeostatic interpretation comes from recovery-sleep paradigms following prolonged wakefulness. In these studies, sleep deprivation normally produces a rebound increase in low-frequency NREM EEG power, reflecting accumulated sleep need. Caffeine consistently attenuated this rebound. In one of the central mechanistic studies, caffeine administered during 40 h of wakefulness reduced waking theta accumulation and, during recovery sleep, significantly reduced NREM EEG power in the 0.75–2.0 Hz range while increasing activity in the 11.25–20.0 Hz range [10]. This pattern indicates that caffeine did not merely stimulate subjects during wakefulness; it also reduced the subsequent electrophysiologic expression of sleep pressure during sleep [10].

Related studies confirmed this result. In genetically stratified samples undergoing 40 h of wakefulness, caffeine reduced recovery-night low-delta power and increased high-alpha/low-sigma activity, again indicating reduced sleep intensity [30]. Another study found that both haplotype groups showed the expected SWA rebound after sleep deprivation. Still, caffeine suppressed this rebound only in non-HT4 carriers of an ADORA2A haplotype, showing that the canonical homeostatic EEG response to caffeine can be abolished in some genetic subgroups [34]. Similarly, caffeine-sensitive and caffeine-insensitive men both showed reduced recovery of low-delta activity after caffeine, though genotype differences emerged more strongly in beta activity than in delta itself [30].

Not all recovery paradigms used full-laboratory spectral PSG, but when EEG-derived metrics were available, the same direction of effect was observed. In a total sleep deprivation study using a Dreem headband, placebo recovery sleep showed the expected increase in slow and delta power, whereas caffeine attenuated delta-band recovery power during NREM and reduced deep-sleep recovery [12]. In operational sleep-loss paradigms, high-dose caffeine similarly blunted the expected early recovery rebound in slow-wave sleep, particularly during the first part of recovery sleep, when homeostatic pressure would normally be strongest [16]. Taken together, these findings strongly support the view that caffeine reduces the EEG expression of sleep homeostasis, especially after prolonged wakefulness [10,12,16,30,34].

#### 3.4.3. Increases in Sigma, Spindle, Beta, and Other Faster Frequencies

A second recurring EEG effect is an increase in faster-frequency activity, especially in the spindle/sigma and beta ranges. In the classic bedtime 100 mg study, caffeine slightly enhanced power in the spindle range (around 12–15 Hz), particularly in central and parietal derivations, while suppressing low-delta power [14]. Morning caffeine likewise increased spindle-frequency activity during NREM sleep, especially in the 11.25–14.0 Hz range and particularly in the first three NREM episodes [15]. In recovery sleep after deprivation, caffeine increased power in the 11.25–20.0 Hz range while reducing the low-frequency rebound, again suggesting lighter and less synchronized sleep [10].

Studies with broader topographic analyses also showed increased activity at higher frequencies. Evening caffeine increased beta-range power, especially in frontal and central regions, while reducing low-frequency power [22]. A larger dose-by-age study similarly found increased power in the 14–19 Hz and 27–32 Hz ranges [23]. Daytime recovery sleep after sleep deprivation also showed caffeine-related increases in 14–19 Hz activity, interpreted as reduced NREM synchronization and a more activated EEG pattern [17].

The most specific faster-frequency finding may be the genotype-dependent increase in beta power reported in the ADORA2A study. There, caffeine-induced beta enhancement in non-REM sleep was largest in C/C carriers, intermediate in C/T, and absent in T/T, suggesting that beta activity may index an insomnia-like cortical arousal response in caffeine-sensitive genotypes [30]. This is notable because the low-delta suppression was not genotype-specific in that study, whereas the beta response was [30]. Thus, increased faster-frequency activity may capture a distinct aspect of caffeine’s sleep EEG effect: not merely reduced sleep pressure, but enhanced cortical arousal or lighter sleep physiology.

An exception to the general sigma-enhancement pattern comes from repeated daytime-use studies. In habitual caffeine users tested 8 h after the last dose, standard PSG sleep architecture was unchanged. Still, sigma activity during NREM was reduced in both the caffeine and withdrawal conditions compared with placebo [33]. This suggests that under chronic daytime exposure, microstructural adaptations may differ from the acute sigma increases observed after bedtime or near-bedtime caffeine [33].

#### 3.4.4. Effects on REM Sleep EEG and REM Timing

Compared with NREM findings, caffeine effects on REM sleep EEG were less consistent and generally smaller, but they were not absent. In some acute nocturnal studies, REM spectra were largely unchanged, with only narrow-band effects or no clear REM differences [14,20]. In contrast, morning caffeine reduced REM sleep EEG power in the 0.75–4.5 Hz and 5.25–6.0 Hz ranges later that night [15]. In the complexity study, REM showed weaker caffeine effects than NREM overall, with the most reliable REM spectral effect being a reduction in theta power [11].

Daytime recovery paradigms suggest that REM may be more vulnerable when sleep occurs under circadian wake pressure. In daytime recovery sleep after total sleep deprivation, caffeine reduced REM sleep. It altered the distribution of wakefulness and REM across the later parts of the sleep episode, especially in the daytime recovery group compared with the normal night-sleep group [18]. Another repeated daytime-use study, in which the main sleep episode was scheduled near the circadian peak of REM promotion, found that caffeine prolonged REM latency and delayed REM accumulation across the sleep episode without significantly changing total REM percentage [35]. Importantly, this occurred without differences in NREM delta power, suggesting that, under this paradigm, caffeine may alter circadian REM regulation more than sleep homeostatic intensity [35].

Age may also moderate REM-related EEG effects. In the entropy/criticality study, caffeine effects in REM were significant mainly in the younger group. In contrast, middle-aged adults showed placebo-night REM EEG patterns already resembling the caffeine-shifted state seen in younger adults [11]. Thus, REM appears less sensitive than NREM overall, but it may still reveal caffeine effects under circadian challenge, in younger adults, or when using more advanced dynamical measures [11,35].

#### 3.4.5. Increased EEG Complexity, Entropy, and Wake-like Dynamics

The newer literature expands the caffeine EEG phenotype beyond conventional spectral power. One study showed that caffeine increased multiple measures of EEG complexity and entropy during sleep, including spectral entropy, sample entropy, spectral sample entropy, and Lempel–Ziv complexity, with effects strongest in NREM sleep [11]. At the same time, caffeine reduced the DFA scaling exponent. It flattened the aperiodic slope, which the authors interpreted as a shift toward a more critical, excitation-dominant, and wake-like neural regime [11]. Spectrally, this was accompanied by reductions in delta, theta, and alpha power and increases in beta power during NREM [11].

These findings are important because they suggest that caffeine not only suppresses slow waves but may also reorganize the overall dynamical structure of sleep EEG. In other words, caffeine appears to move sleep away from a highly synchronized, low-frequency-dominated state and toward a more information-rich, desynchronized, and wake-like cortical state [11]. This interpretation aligns well with earlier spectral findings of reduced delta and increased faster-frequency power [10,14,22], but provides a broader framework for understanding caffeine’s effects on sleep-brain dynamics.

#### 3.4.6. Contextual Modifiers: Dose, Timing, Age, Habitual Use, and Individual Differences

Although the direction of effect was fairly consistent, the magnitude and expression of caffeine’s sleep EEG effects depended on several modifiers. Dose and timing were important: low bedtime doses such as 100 mg were sufficient to reduce early-night SWA [14], whereas higher doses produced broader disturbances in sleep depth and architecture [23,37,40,41]. Morning caffeine intake can still influence nighttime EEG even at low bedtime concentrations [15]. At the same time, repeated daytime intake sometimes left conventional sleep architecture intact but altered EEG microstructure, especially sigma activity and REM timing [33,35].

Age-altered baseline EEGs were substantial, but age differences in the caffeine response were usually modest. Middle-aged adults generally had lower low-frequency power and poorer sleep overall. Yet, most studies did not find major age-by-caffeine interactions on the core low-frequency EEG response [17,22,23]. More subtle age effects did appear in specific REM and higher-frequency measures [11,22].

Habitual intake and tolerance also mattered. Some studies reported that habitual caffeine use did not moderate sleep disruption to any significant extent [16]. In contrast, others found that higher daily intake predicted worse recovery-night sleep continuity or shorter recovery sleep after sleep deprivation [12]. Repeated-use studies suggest that tolerance may develop for some macrostructural effects, even as EEG microstructure continues to change [33,35]. Relatedly, withdrawal increased sleep pressure and sleep ability in one circadian-lab study, but ongoing daytime caffeine use itself did not measurably alter melatonin timing or many standard sleep variables [27].

Finally, individual biological vulnerability clearly modifies caffeine’s effects on the EEG. ADORA2A genotype influenced the size of caffeine-induced beta activation during recovery sleep [30] and determined whether caffeine could suppress the SWA rebound after sleep deprivation [34]. Subjective caffeine sensitivity was also associated with differential EEG topography during waking and sleep, particularly frontocentral versus parieto-occipital low-frequency activity [36]. Beyond genetics, one study showed that normal sleepers with high stress-related sleep reactivity were much more vulnerable to caffeine-induced sleep-initiation disruption, with a trend toward reduced slow-wave sleep as well [39]. These results indicate that caffeine’s effect on sleep EEG is not uniform across individuals, but depends partly on adenosinergic biology and trait-like sleep vulnerability [30,34,36,39].

### 3.5. Change in SWA/Delta Power (%, or Nearest Reported Proxy)

Where quantified, caffeine-related suppression of low-frequency sleep EEG was often substantial. For example, in recovery sleep after sleep deprivation, high-dose caffeine and third-sleep-wave first-third slow-wave sleep from about 75 to 40 min (≈47% reduction) in one study [16]. In late middle-aged adults, bedtime caffeine reduced slow-wave sleep in the first 3 h from 66 to 38 min (≈42% reduction) [19]. In adolescents, evening caffeine reduced SWS proportion from 40.5% to 36.9%, corresponding to a 3.6-percentage-point or ≈9% relative reduction [25]. In a fixed-duration sleep opportunity, caffeine reduced stage 4 sleep from 15.6% to 9.8% (≈37% reduction) [28]. In a home EEG study, 400 mg caffeine reduced slow-wave sleep from 71.5 min to 56.7 min at bedtime (≈21% reduction) and to 48.9 min when taken 6 h before bedtime (≈32% reduction) [37]. Other studies clearly showed suppression of low-frequency NREM activity but did not report a single percentage reduction that could be extracted directly [10,13,14,15,17,18,22,23,30,33,35].

### 3.6. Risk of Bias Assessment

The bias risk assessment is presented in Table 4 (Rob-2) and Table 5 (ROBINS-I).

## 4. Discussion

The sleep-related EEG outcomes converge on a coherent phenotype: caffeine exposure is associated with reduced NREM low-frequency activity (including the slowest delta ranges often used as proxies for sleep pressure), relative increases in faster frequencies usually linked to arousal, and greater fragmentation or reduced consolidation in contexts that already challenge sleep (e.g., recovery sleep after prolonged wakefulness, or daytime recovery sleep). In several paradigms, these microstructural changes occurred alongside modest but measurable macrostructural disruption (longer sleep latency, reduced total sleep time, and/or deep sleep). In contrast, subjective sleep quality did not consistently track objective disturbance—an observation consistent with broader caffeine-sleep literature showing imperfect self-perception of caffeine’s sleep impact.

A second cross-study regularity is state dependence: caffeine effects were most evident when homeostatic sleep pressure or circadian wake promotion was high (e.g., after sleep deprivation, or when sleep was scheduled into the biological day), implying that caffeine does not merely add generic activation, but interacts explicitly with the physiology that normally deepens and stabilizes NREM sleep [42].

Finally, the EEG results indicate substantial inter-individual variability (including age- and habit-related differences), which aligns with well-documented variability in caffeine pharmacokinetics and pharmacodynamics (half-life ranging widely across individuals, receptor/genetic differences, tolerance/withdrawal effects) [43].

### 4.1. Mechanistic Interpretation

Caffeine’s mechanistic explanation for the observed EEG phenotype is strongest when built from three layers of non-EEG evidence: (i) adenosine as a mediator of sleep homeostasis, (ii) receptor pharmacology and in vivo receptor occupancy at typical doses, and (iii) downstream neuromodulatory and systems-level effects that bias the brain toward arousal and desynchronization.

#### 4.1.1. Adenosine as a Sleep–Wake Homeostatic Signal

Adenosine has long been supported as a candidate mediator of the sleep-inducing effects of prolonged wakefulness. In vivo microdialysis work showed that extracellular adenosine in the basal forebrain rises during wake, increases progressively during sustained wakefulness, and declines during recovery sleep; experimentally increasing extracellular adenosine in the basal forebrain can mimic aspects of the post-wake sleepiness/sleep profile [42]. Critically for the interpretation of EEG findings, these results imply that the neural substrate that tracks time awake is neuromodulatory and metabolic, not merely an emergent property of cortical oscillations—so blocking adenosine signaling should weaken the slow-wave expression typically driven by high homeostatic pressure, consistent with the low-frequency suppression repeatedly observed in the sleep-related EEG findings.

An additional implication of this framework is that slow-wave suppression by caffeine may signify more than simply “lighter” sleep. Convergent theoretical and experimental work suggests that slow-wave activity reflects a core homeostatic mode of cortical synchronization linked to prior waking plastic load and to overnight synaptic renormalization. In this view, the progressive decline of slow-wave activity across the night is not merely a passive spectral phenomenon, but part of a biologically meaningful recalibration of synaptic strength after wakefulness. Accordingly, caffeine-related attenuation of low-frequency sleep EEG may be interpreted as a weaker expression of the synchronized cortical regime through which the brain discharges typical sleep pressure and reorganizes waking-induced plastic changes [44,45].

#### 4.1.2. Receptor Pharmacology and In Vivo Occupancy at Typical Doses

At doses commonly consumed by humans, caffeine’s dominant mechanism is competitive antagonism at adenosine receptors, especially A1 and A2A subtypes (with “high-dose” mechanisms such as phosphodiesterase inhibition requiring substantially higher concentrations than typical dietary intake) [46]. Importantly, this is not a purely in vitro claim: human PET imaging demonstrates that caffeine can occupy a substantial fraction of cerebral adenosine receptors at behaviorally relevant exposures. In a PET occupancy study, Elmenhorst and colleagues quantified caffeine occupancy of human A1 receptors in the cerebral cortex. They concluded that, given caffeine’s biological half-life, repeated daily consumption could maintain sizable A1 receptor occupancy [47]. Complementarily, Ishibashi and colleagues used PET to estimate dose–occupancy relationships for striatal A2A receptors, reporting that a “cup of coffee” scale dose (~100 mg caffeine) can exceed the estimated ED50 for A2A receptor occupancy, implying substantial blockade in typical coffee consumers [48].

These imaging data strengthen the causal interpretability of the EEG phenotype in two ways. First, they support that the doses used in many sleep studies (including modest doses) plausibly reach receptor occupancy levels sufficient to meaningfully alter network excitability during sleep onset and early NREM, when sleep intensity commonly peaks [47]. Second, they provide a mechanistic bridge between “residual caffeine level” and physiological impact: even as peripheral concentrations decline, receptor occupancy and downstream effects may remain substantial, particularly in slow metabolizers or with repeated dosing [43].

A further mechanistic consideration is that the sleep-relevant biological effect of repeated caffeine use is not determined by caffeine. In humans, caffeine is metabolized predominantly by hepatic CYP1A2 [49], and approximately 80–85% of the dose is converted to paraxanthine, the principal dimethylxanthine metabolite [50]. After a single caffeine exposure, paraxanthine rises more slowly than parent caffeine [51]. exceed that of caffeine approximately 8–10 h after intake [52], which is directly relevant to sleep when caffeine is consumed throughout the morning or repeatedly across the day. Thus, the biologically important purinergic burden present at sleep onset may reflect not only residual caffeine but also delayed and persistent exposure to paraxanthine.

This point is mechanistically important because paraxanthine is not an inert breakdown product. Like caffeine, paraxanthine acts as an adenosine receptor antagonist [53] and is roughly equipotent to caffeine at adenosine receptors, with substantial wake-promoting and sympathomimetic activity [54]. Human pharmacology studies indicate that paraxanthine can reproduce key stimulant-like physiological effects [55], and animal sleep studies further suggest that paraxanthine itself promotes wakefulness and suppresses both NREM and REM sleep [56]. Therefore, from a sleep-EEG perspective, paraxanthine should be viewed as an active continuation of methylxanthine signaling rather than merely a marker of prior caffeine exposure.

The relevance of paraxanthine is likely greatest under repeated daily intake. Experimental human work using a typical repeated-consumption schedule (150 mg caffeine three times daily for 10 days) showed that both caffeine and paraxanthine remained elevated overnight, and that paraxanthine could remain elevated even after 24–36 h of abstinence [50]. These findings imply that daily caffeine use may create a carryover state in which adenosinergic antagonism persists into the biological night even when bedtime caffeine concentrations appear modest. In practical terms, apparent “daytime-only” caffeine use may still influence nocturnal sleep physiology because the combination of residual caffeine and accumulated paraxanthine may prolong effective receptor blockade beyond what would be inferred from caffeine alone.

This combined caffeine–paraxanthine exposure provides a plausible framework for understanding why repeated intake can alter sleep homeostasis even in the absence of large bedtime caffeine levels. Sleep homeostasis depends strongly on adenosine signaling during prolonged wakefulness [42] and on the subsequent expression of low-frequency synchronized activity during NREM sleep. If both caffeine and paraxanthine continue to antagonize adenosine receptors across the day and into the night, the build-up, transmission, and expression of homeostatic sleep pressure may be blunted at multiple levels. This could help explain why repeated caffeine intake and recovery-sleep paradigms often show attenuated slow-wave activity, reduced low-delta rebound, and relatively greater faster-frequency activity: the homeostatic system may be entering sleep under conditions of persistent purinergic antagonism rather than true neurochemical recovery.

At present, direct human evidence specifically isolating paraxanthine’s independent contribution to sleep EEG remains limited. Most sleep studies measured caffeine exposure at the protocol level rather than simultaneously quantifying caffeine, paraxanthine, receptor occupancy, and overnight EEG outcomes. Accordingly, the available evidence supports a cautious but biologically coherent interpretation: repeated caffeine intake may influence sleep EEG through the combined, temporally extended action of caffeine and paraxanthine, whereas the precise independent and synergistic contributions of each compound remain insufficiently resolved. Future sleep-EEG studies should therefore incorporate serial pharmacokinetic sampling of both caffeine and paraxanthine, particularly in repeated-intake designs, to determine whether metabolite accumulation explains inter-individual differences in slow-wave suppression, sleep fragility, and apparent tolerance.

#### 4.1.3. Downstream Neuromodulation and Systems-Level Arousal Bias

Blocking adenosine signaling removes an inhibitory constraint on multiple arousal-related neurotransmitter systems (cholinergic, dopaminergic, glutamatergic, and, indirectly, noradrenergic and others), shifting the brain toward a more activated state. Evidence for such downstream changes comes from neurochemical and neuroimaging studies that do not depend on sleep EEG microstructure.

In vivo microdialysis and pharmacologic studies indicate that caffeine can enhance cholinergic signaling via A1 receptor mechanisms. Carter and colleagues reported that caffeine enhanced hippocampal acetylcholine release in vivo, consistent with the view that endogenous adenosine exerts tonic inhibitory control over cholinergic neurotransmission via A1 receptors, and that caffeine disinhibits acetylcholine release [57]. Because acetylcholine is tightly linked to cortical activation and reduced slow-oscillatory synchronization, this provides a plausible neurochemical pathway from adenosine blockade to reduced NREM slow-wave expression and increased fast activity in the sleep-related EEG.

Similarly, Solinas and colleagues demonstrated that behaviorally relevant doses of caffeine increased extracellular dopamine and glutamate in the nucleus accumbens shell, implicating adenosine receptor blockade as a permissive step for arousal- and motivation-related neuromodulation [58]. Although dopamine/glutamate changes are not themselves EEG measures, they predict a network-level shift away from bistable, highly synchronized cortical dynamics toward more irregular and activated states—qualitatively consistent with reduced low-frequency power and increased complexity/fast activity reported in the review’s outcomes.

Human neuroimaging provides convergent systems-level evidence. Volkow and colleagues used PET to show that caffeine (300 mg, orally) increased D2/D3 receptor availability in striatal regions and that these changes were associated with increased alertness [59]. Resting-state fMRI work also suggests that caffeine/coffee intake modulates large-scale network connectivity in ways consistent with increased attentional engagement and altered default-mode coupling, even while vascular effects complicate interpretation of BOLD signals [60]. Together, these findings support the idea that caffeine biases the brain toward attentional/arousal network configurations, offering a plausible systems-level substrate for the wake-like EEG shifts observed during sleep.

Finally, expectancy effects can modulate arousal-related neurochemistry and may act as an underappreciated methodological confound in caffeine–sleep studies. Kaasinen and colleagues reported that expectation of caffeine (placebo) induced dopaminergic responses and correlated with arousal, indicating that cognitive context can recruit some of the same pathways as caffeine itself [61]. This implies that blinding integrity and participant expectations are nontrivial for interpreting modest sleep EEG effects—especially when subjective sleep disruption is weak or absent.

### 4.2. Sleep Architecture and Homeostasis

The EEG phenotype strongly aligns with macrostructural sleep effects documented in the broader polysomnography and sleep-monitoring literature: caffeine delays sleep initiation, shortens sleep duration, reduces sleep efficiency, increases wake after sleep onset, and shifts stage composition away from deep sleep [62]. In a systematic review and meta-analysis, caffeine consumption reduced total sleep time and sleep efficiency, increased sleep onset latency and wake after sleep onset, increased light sleep (N1), and decreased deep sleep (N3/N4) [62]. These effects provide a macrostructural scaffold that can “explain” part of the EEG spectral shifts: if caffeine increases time in lighter NREM stages and reduces time in deep NREM, the net NREM spectrum will shift toward lower slow-wave power and potentially relatively higher sigma/beta components, even without invoking direct effects on oscillation generators.

However, the sleep-related EEG outcomes suggest that caffeine’s influence extends beyond stage redistribution to altered within-stage dynamics—particularly early in sleep and under high sleep pressure. This pattern is mechanistically coherent with adenosine-based homeostasis: adenosine accumulation with wakefulness externalizes “Process S” (sleep pressure) into a biochemical signal, and antagonizing its receptors should weaken the expression of the deepest, most homeostatically driven components of NREM sleep. The observation that caffeine effects can appear disproportionately when sleep pressure is most significant (e.g., after prolonged wakefulness) is consistent with a model in which adenosinergic tone is higher in those states, thereby giving caffeine a larger “functional leverage” even at similar plasma concentrations.

Timing effects observed in polysomnography studies further constrain interpretation. Drake and colleagues showed that a fixed caffeine dose (400 mg) disrupted sleep even when administered 6 h before bedtime, supporting the idea that “behaviorally meaningful” caffeine exposure can persist across a typical evening and still affect nocturnal sleep initiation and maintenance. This aligns with the EEG results, where alterations were frequently most substantial in early sleep (when both residual caffeine and maximal homeostatic drive coincide) and helps explain why even “morning” or “daytime” caffeine exposures might be linked to nocturnal microstructure effects in some designs: variability in clearance and repeated intake can maintain receptor occupancy into the night.

In this framework, the EEG pattern can be interpreted as a physiological signature of impaired sleep recuperation: less slow-wave activity implies reduced expression of the very feature (deep synchronized NREM) most closely associated with high sleep pressure and restoration. While “EEG restoration” claims must be made cautiously, the convergence of (i) adenosine as a wake-accumulating sleep factor and (ii) caffeine’s receptor blockade/occupancy supports a mechanistically grounded account of why low-frequency NREM markers are consistently suppressed in the sleep-related EEG.

Another factor relevant to interpretation is that tolerance to caffeine appears to be incomplete and domain-specific. In controlled human work, repeated caffeine exposure produced greater adaptation in subjective measures than in physiological indices, with evidence that acute abstinence increased waking EEG theta and altered cerebral blood-flow measures even when users may have perceived themselves as largely adapted [63]. This dissociation is clinically significant because it suggests that habitual consumers may underestimate the extent to which caffeine continues to influence sleep-relevant neurobiology. Thus, a weak subjective perception of sleep disruption should not be taken as evidence that nocturnal sleep physiology has normalized under regular caffeine use [63,64].

This subjective–physiological dissociation may help explain why many participants do not report marked sleep disturbance even when sleep EEG shows a more aroused cortical state. Caffeine can help maintain a sense of alertness, normality, or “acceptable sleep.” At the same time, the underlying NREM physiology shifts toward reduced slow-wave expansivity, with relatively greater sigma/beta activity, a pattern more consistent with lighter, restorative sleep. In other words, the sleeping brain may remain electrophysiologically activated even when the person does not consciously perceive substantial disruption. This mismatch is clinically important because it suggests that self-reported sleep quality may underestimate caffeine’s neurobiological impact, particularly among habitual users who develop partial tolerance to some subjective effects but not to microstructural EEG changes. From a practical perspective, this means that the absence of a strong subjective complaint should not be interpreted as evidence that caffeine is harmless for sleep physiology; quantitative EEG may reveal a persistent burden on cortical recovery processes that conventional sleep-stage summaries or self-report alone can miss.

An additional point is that the biologically relevant adenosine antagonism at bedtime may not be determined due to caffeine. Caffeine’s major metabolite, paraxanthine, also has substantial adenosine receptor–blocking properties and can remain elevated even when parent caffeine concentrations have fallen to relatively low levels. This is especially relevant for morning or repeated daytime intake, because the apparent absence of high bedtime caffeine does not necessarily mean that adenosinergic signaling has fully normalized before sleep. Instead, persistent paraxanthine exposure may help sustain a degree of receptor antagonism into the night, providing a plausible explanation for why morning caffeine administration can still alter nocturnal EEG microstructure, including reduced low-frequency NREM activity and relatively greater faster-frequency activity. Clinically and experimentally, this means that bedtime caffeine concentration alone may underestimate the true carryover effect of daytime caffeine on the sleeping brain.

### 4.3. Circadian Interactions

The EEG results indicate that caffeine’s sleep-disrupting effects are amplified when sleep occurs at a circadianly unfavorable time (e.g., daytime recovery sleep) or when the circadian wake drive rises across the sleep attempt. Non-EEG circadian evidence provides two complementary explanatory routes. First, caffeine can directly shift the circadian clock. Burke and colleagues showed that evening caffeine (dose equivalent to a double espresso, administered 3 h before habitual bedtime) delayed the circadian melatonin rhythm by ~40 min, with mechanistic evidence implicating adenosine receptor/cAMP-related pathways [65]. Even a ~40-min phase delay is biologically meaningful: it implies that sleep is initiated at a relatively “earlier” internal circadian phase than intended, increasing circadian wake-promoting pressure at sleep onset and potentially lowering the ease with which the brain transitions into stable deep NREM. This makes the sleep EEG phenotype (less synchronized, more fragmented) more likely, especially when caffeine is taken in the late afternoon/evening.

Second, even without a substantial phase shift, caffeine may function as a “circadian gate opener” by reinforcing wake-promoting circuits at times when the circadian system already promotes wake. In practice, this means that caffeine can be disproportionately disruptive to daytime sleep (or late-night/early-morning sleep in shift workers), not merely because of pharmacology but because it adds to a biological context that resists sleep.

Genetic-epidemiologic work nuances the circadian story. A Mendelian randomization analysis reported limited evidence for causal relationships between lifetime-average caffeine intake and sleep traits, suggesting that many observational associations may reflect shared environments (stress, workload) rather than direct causation—while still acknowledging robust acute effects when caffeine is taken near bedtime [66]. This distinction is highly relevant for interpreting EEG studies: sleep EEG experiments typically test acute, time-specific exposures under controlled conditions, whereas genetic instruments index long-run consumption patterns that average across timing and tolerance. Therefore, null or weak causal inference at the “habitual intake” level is not inconsistent with substantial acute, circadian-phase-dependent impacts on sleep physiology.

A deeper mechanistic interpretation can be made by viewing caffeine’s circadian effects within the framework of the two-process model of sleep regulation. In that model, sleep propensity at any given time is determined by the interaction between a homeostatic process that rises with time awake and declines during sleep, and a circadian process that rhythmically promotes wakefulness or sleep depending on biological day. Caffeine is especially disruptive when these two processes are in opposition—namely, when homeostatic sleep pressure is high, but the circadian system is simultaneously promoting wakefulness. This helps explain why caffeine often has a more pronounced effect during daytime recovery sleep after sleep deprivation than during habitual nocturnal sleep: the individual attempts to sleep under strong homeostatic pressure, but at a circadian phase that actively resists sleep initiation and consolidation. Under these conditions, even a modest additional reduction in adenosine-mediated sleep drive may shift the balance toward lighter, less stable, and less synchronized sleep.

This interaction is likely not merely behavioral, but also mechanistically linked to the molecular circadian clock. The core cellular clock is organized around transcriptional–translational feedback loops involving CLOCK and BMAL1, which drive the rhythmic expression of PER and CRY genes and thereby generate approximately 24 h oscillations in cellular physiology [67,68]. Available evidence suggests that caffeine can influence this molecular timing system indirectly through adenosine receptor–dependent signaling [65]. Human in vivo work has shown that evening caffeine delays the circadian melatonin rhythm [65], while in vitro experiments demonstrated that caffeine lengthens the period of molecular circadian oscillations through a mechanism involving adenosine receptor antagonism and cyclic-AMP-related signaling [65]. Thus, caffeine should be considered not only a wake-promoting compound, but also a potential modulator of circadian clock timing and clock-controlled physiology.

Adenosine provides a plausible bridge between sleep homeostasis and circadian timing. From the homeostatic perspective, adenosine accumulates during prolonged wakefulness and contributes to the build-up of sleep pressure, particularly the propensity for synchronized low-frequency NREM activity. From a circadian perspective, adenosine signaling also interacts with the circadian pacemaker and can modify the clock’s sensitivity to entraining signals. Experimental work has shown that adenosine receptor pathways can integrate sleep-loss-related signals with circadian timing mechanisms, supporting the idea that caffeine may weaken homeostatic sleep pressure while also modifying circadian phase regulation or circadian gating. In this model, caffeine’s stronger disruption of daytime recovery sleep is not explained simply by “more wakefulness.” Still, by a dual action: attenuation of homeostatic sleep drive together with reinforcement or disinhibition of circadian wake-promoting influence.

This framework also helps clarify why daytime recovery sleep is especially vulnerable at the EEG level. After prolonged wakefulness, homeostatic pressure would normally favor rapid sleep onset, strong early-night-like low-frequency synchronization, and a rebound in slow-wave activity. However, if sleep is attempted during the biological day, the circadian system continues to promote alertness and opposes stable sleep. Caffeine appears to widen this mismatch further. By antagonizing adenosine receptors, it reduces the physiological expression of sleep pressure, and by influencing circadian timing pathways, it may shift or reinforce a wake-favoring internal state. The consequence is a quantitatively steeper imbalance between Process S and Process C: high prior wake time is no longer sufficient to overcome circadian wake promotion to the same extent, and the EEG therefore shows reduced low-frequency power, more high-frequency activity, poorer consolidation, and a weaker homeostatic rebound than would otherwise be expected.

At present, however, direct human evidence linking caffeine-induced sleep EEG changes to CLOCK- or BMAL1-specific alterations in vivo remains limited. Therefore, the most defensible interpretation is that current human and translational evidence support an abiologically plausible clock-related mechanism, rather than a fully resolved gene-specific causal pathway. Future sleep-EEG studies should combine quantitative EEG with circadian phase markers, repeated pharmacokinetic sampling, and, where feasible, molecular or genetic circadian phenotyping, to test more directly how caffeine modifies the interaction between sleep homeostasis and circadian timing.

### 4.4. Effects on Complexity

The physiological significance of increased sleep EEG complexity is not straightforward. In general, deep non-rapid eye movement sleep is characterized by strong neuronal synchronization, recurrent up- and down-state alternation, and prominent low-frequency oscillations, especially slow waves [69,70]. These dynamics produce a more stereotyped and temporally regular signal, which is typically associated with lower complexity than wakefulness or REM sleep [71]. By contrast, higher complexity or entropy during sleep usually indicates that cortical activity has become less globally synchronized, less dominated by stable slow oscillations, and more variable across time, thereby resembling a more activated or wake-like network regime [72].

From this perspective, caffeine-related increases in complexity and entropy are physiologically compatible with the broader pattern seen across the sleep EEG literature. The most robust electrophysiological effect of caffeine in this review is suppression of low-frequency NREM activity, particularly slow-wave activity and the lowest delta frequencies, often accompanied by relative enhancement of faster-frequency activity. Because slow-wave activity is a classic marker of homeostatic sleep pressure and synchronized cortical recovery dynamics, a shift toward greater EEG complexity can be interpreted as the dynamical counterpart of reduced NREM synchronization. In other words, the signal becomes more irregular not because sleep is “richer” in a restorative sense, but because the brain is less stably engaged in the highly coordinated oscillatory regime that normally characterizes deep restorative sleep.

This interpretation is also relevant to synaptic plasticity and sleep function. Slow oscillations and their coordination with other sleep rhythms are thought to provide a temporal framework for synaptic renormalization, memory processing, and large-scale neuronal recovery during sleep [70,73]. Accordingly, when caffeine increases complexity while simultaneously reducing low-frequency synchronization, the most plausible implication is not improved sleep-related information processing, but rather partial destabilization of the network conditions that normally support sleep-dependent restoration. In this sense, increased complexity during caffeine-exposed sleep may reflect the persistence of a more excitable cortical state into sleep, with reduced expression of the coordinated low-frequency activity typically associated with biological recovery.

The relationship between complexity and classic EEG indicators should therefore be understood as complementary rather than redundant. Slow-wave activity quantifies the intensity of synchronized low-frequency oscillations [74], whereas complexity measures describe how predictable, regular, or structured the signal is across time and scales [71,75]. These metrics are related because stronger slow-wave dominance generally corresponds to a more stereotyped and less entropic signal, but they are not interchangeable [76,77]. Complexity may capture dynamical aspects of sleep organization that are not fully reflected by spectral power alone, especially when caffeine shifts the brain toward lighter sleep, increased excitation, and altered large-scale network criticality. For this reason, complexity findings strengthen rather than replace the traditional interpretation based on slow-wave suppression and wake-like intrusion into NREM sleep.

At present, however, the evidence remains limited and should be interpreted cautiously. The complexity findings come based on a small number of recent studies, and direct links between caffeine-induced changes in complexity and next-day recovery, memory consolidation, or other functional outcomes have not yet been fully established. Thus, the most defensible conclusion is that increased complexity and entropy under caffeine likely reflect reduced cortical synchronization and a less restorative sleep state. Still, additional work is needed to determine how strongly these dynamical changes predict impaired recovery or altered sleep-dependent plasticity.

### 4.5. Behavioral and Clinical Implications

The EEG phenotype is not merely a laboratory curiosity: it corresponds to a biologically and behaviorally meaningful trade-off between acute alertness gains and subsequent sleep costs. Controlled field research indicates that caffeine can maintain vigilance and operational performance during sustained wakefulness, consistent with its widespread use in military and shift-work contexts. McLellan reported that caffeine improved vigilance and performance metrics during prolonged wakefulness in Special Forces personnel, supporting a functional arousal benefit that is likely mediated by adenosine blockade and the neuromodulatory pathways discussed above [78]. Yet the exact adenosine-blockade mechanisms that preserve performance under sleep loss predict impaired recovery sleep depth—precisely the pattern reflected in reduced low-frequency NREM activity and greater wake-like features in the EEG.

Clinically, the most defensible translation is not “avoid caffeine” but “treat timing and dose as sleep-relevant exposures.” The best-supported implication of sleep hygiene is that caffeine can disrupt sleep, even when used well before bedtime. In controlled testing, caffeine at 6 h pre-bedtime still produced significant sleep disruption, and meta-analytic evidence indicates meaningful average reductions in total sleep time and deep sleep [62]. Therefore, EEG evidence of reduced slow-wave components can be interpreted as a microstructural correlate of a macrostructural phenomenon already clinically recognized: late-day caffeine use compromises sleep initiation and depth, and individuals may underestimate the extent of the disruption.

Population studies further contextualize risk in a nationally representative US sample, caffeine consumption related to insomnia symptoms in unadjusted models, but associations attenuated after adjusting for covariates (e.g., anxiety, race/ethnicity), underscoring confounding and the likelihood of bidirectional relationships (poor sleep → caffeine use) [79]. In adolescents, observational studies link higher caffeine intake to shorter sleep duration, with a dose-dependent association, underscoring potential vulnerability in developmental contexts and the public-health salience of caffeine from soft drinks/energy-related sources rather than coffee/tea alone [80]. These epidemiologic findings do not replace controlled physiology. Still, they help interpret why real-world EEG and sleep outcomes can be heterogeneous: timing, reasons for use (fatigue compensation), and co-occurring stress/anxiety can dominate variance.

Finally, individual differences have direct clinical relevance. Genetic association studies show that variation in ADORA2A is associated with subjective caffeine sensitivity and sleep complaints, suggesting that a one-size-fits-all timing recommendation will under-protect a subset of caffeine-sensitive individuals [81]. Meanwhile, pharmacokinetic studies show wide interindividual half-life ranges (driven by CYP1A2 activity, smoking status, medications, and other factors), resulting in substantial variability in the extent of receptor blockade that persists into the sleep period at identical doses [82]. This variability provides a mechanistic account for why the EEG summaries show mixed magnitudes and occasional residual effects even when dosing was earlier in the day. For some individuals, “earlier” does not mean “cleared”.

## 5. The Importance of Caffeine’s Effects on Sleep-Related EEG in the Context of Sport and Sports Research

From a sport-science perspective, caffeine-related changes in sleep EEG are significant not only because they indicate “poorer sleep,” but because they index which physiological recovery processes are being altered (e.g., slow-wave activity, sigma/spindle dynamics, cortical arousal), and these processes are directly relevant to adaptation, motor learning, vigilance, and injury risk. This is a key translational point for sports research: standard sleep variables (total sleep time, sleep efficiency, sleep stages) are often too coarse to detect subtle but functionally meaningful changes in recovery physiology, whereas EEG microstructure can reveal early disturbances in homeostatic recovery even when macrostructure appears relatively preserved.

This matters because athlete performance depends on repeated cycles of training load, neural recovery, and skill consolidation. Across sports, insufficient or disturbed sleep is consistently associated with impaired reaction time, mood, decision-making, and recovery quality, all of which influence training output and competition readiness [83,84,85]. In youth and adolescent athletes, shorter habitual sleep has also been linked to higher injury risk [86], reinforcing that sleep is not merely a wellness variable but a performance and safety variable. Experimental work in athletes similarly shows that extending sleep can improve sprint performance, shooting accuracy, and daytime alertness [87], implying that any intervention that degrades sleep physiology—even if it improves immediate alertness—may carry downstream costs for performance adaptation.

Within this framework, sleep EEG is particularly relevant because it tracks mechanisms that are central to sport recovery. Slow-wave activity (SWA) and low-frequency NREM power are widely interpreted as markers of sleep homeostasis and cortical recovery. In contrast, spindle/sigma activity is implicated in synaptic plasticity and motor memory consolidation [88,89,90,91]. In sport, where technical skill is continuously learned and re-learned, these EEG features are more informative than sleep duration alone. For example, motor sequence learning and visuomotor skill retention have been linked to NREM spindle activity and sleep-dependent plasticity [89,92,93]. Thus, if caffeine shifts sleep EEG toward lower SWA or more high-frequency activity, the concern in sport is not just “sleep loss,” but potentially reduced neural restoration and weaker overnight consolidation of training-related motor adaptations.

This is especially important because many athletes use caffeine strategically in contexts that already challenge sleep homeostasis, such as evening training or competition, travel, jet lag, and sleep restriction. Elite and team-sport athletes frequently report short sleep duration, delayed sleep onset, and fragmented sleep around competition and travel [94,95,96,97]. In these situations, caffeine can acutely preserve alertness and performance capacity, which is why it remains one of the most used ergogenic aids [98,99]. However, the sleep-EEG literature suggests that caffeine may also blunt the electrophysiological expression of recovery sleep, particularly in low-frequency NREM activity. For sport scientists, this raises a practical trade-off: the same intervention that protects performance during wakefulness may partially impair the very sleep physiology needed for subsequent adaptation and readiness.

A more applied sports-science interpretation can be made by considering the contexts in which athletes actually use caffeine. In sport, caffeine is rarely consumed under ideal laboratory conditions; instead, it is often used before late-afternoon or evening training, before night competition, on repeated competition days, and during or after transmeridian travel. These are precisely the situations in which sleep is already vulnerable because of elevated arousal, delayed post-exercise wind-down, competition stress, irregular schedules, or circadian misalignment. Therefore, the practical significance of the present sleep-EEG findings is not simply that caffeine “can disturb sleep,” but that it may do so in athletes at the exact moments when sleep is most needed for recovery and subsequent performance.

Evening competition and late training appear especially relevant. Recent athlete-focused review indicates that evening caffeine intake in the approximate range of 3–6 mg/kg is commonly associated with poorer subsequent sleep, with the most consistent findings involving reduced subjective sleep quality and, in several studies, reductions in total sleep time and sleep efficiency together with longer sleep latency. Importantly, objective findings in athletes are more mixed than subjective reports, and the athlete-specific literature remains methodologically limited [100]. Direct evidence on sleep EEG in athletes is particularly sparse: most studies have used questionnaires or actigraphy, and only a very small number have employed polysomnography. Thus, the athlete literature does not yet provide a robust sport-specific EEG map of caffeine effects. However, the available evidence is directionally consistent with the broader human sleep literature. It supports the concern that evening ergogenic caffeine use may come at a cost to recovery, even when next-day performance impairment is not immediately obvious.

This distinction has practical importance for dose selection. In athlete studies, lower evening doses do not always produce measurable objective sleep disruption, whereas higher doses, especially around 6 mg/kg, more often impair sleep quality, sleep efficiency, or time asleep [101,102]. From an applied standpoint, this suggests that caffeine recommendations in sport should not be framed only in terms of ergogenic efficacy, but also in terms of the recovery burden imposed by timing and dose. For athletes competing or training late in the day, the key question is not whether caffeine can improve acute performance, but whether the expected performance gain justifies the likely reduction in overnight recovery quality, particularly when events occur on consecutive days.

A second practically important scenario is repeated caffeine use across congested competition schedules or in response to accumulated fatigue. In these settings, athletes may consume caffeine repeatedly to maintain alertness, reaction time, or motivation, while the sleep opportunity between events is already shortened. The present review suggests that such use may be especially problematic from a recovery-EEG perspective, because caffeine can attenuate low-frequency NREM activity and weaken the electrophysiological expression of recovery sleep. In sport, this raises the possibility that repeated caffeine intake may preserve short-term output while progressively impairing the neural recovery processes that support later technical execution, decision-making, and adaptation. This concern is amplified when athletes use caffeine on successive evenings or use a second dose later in the day to compensate for earlier fatigue.

A third athlete-specific issue concerns travel and jet lag. Athletes commonly use caffeine to counter daytime sleepiness and maintain performance after travel, and this may be useful when timed appropriately. However, travel-related circadian disruption already places sleep in a fragile state, particularly after eastward travel or when sleep is attempted at a biologically adverse time. In that context, poorly timed caffeine may exacerbate the mismatch between circadian phase and homeostatic sleep need. The practical implication is that caffeine during travel should be treated as a circadian tool rather than simply an ergogenic stimulant: morning use at the destination may help alertness, whereas late-day or evening use may delay adaptation and further compromise post-travel sleep. For athletes facing rapid travel followed by training or competition, the trade-off is not only between performance and sleep, but also between immediate alertness and the speed of circadian realignment.

A further sport-specific concern is that caffeine may be used not only to enhance performance acutely, but also to mask fatigue that is partly a consequence of prior caffeine-related sleep disruption. In this scenario, the athlete may feel functionally “rescued” the next day because caffeine restores alertness, vigilance, or motivation. At the same time, the underlying need for neurorehabilitation is met. This creates a potentially self-reinforcing cycle: caffeine disturbs elements of sleep physiology that support restoration and training adaptation; the athlete then uses caffeine again to compensate for the resulting fatigue or reduced readiness; and the immediate performance benefit may obscure the accumulating recovery cost. From a sports-science perspective, this is important because the relevant trade-off is not simply between sleep and wakefulness, but between acute ergogenic gain and the integrity of overnight processes needed for subsequent adaptation, including cortical recovery, synaptic plasticity, and sleep-dependent consolidation of motor learning. Thus, repeated reliance on caffeine in training or competition phases could preserve short-term output while gradually compromising the neurophysiological recovery on which later training quality, learning, and readiness depend.

A third reason caffeine-related sleep EEG effects are essential in sports research is that EEG microstructure may help explain inter-individual variability in “good” versus “bad” responders to caffeine. Athlete responses to caffeine are heterogeneous at the behavioral level (performance gains, side effects, sleep disruption), and part of this variability likely reflects differences in adenosinergic signaling and genetic susceptibility (e.g., ADORA2A-related sensitivity) rather than only dose or timing [103]. Pharmacogenetic and sensitivity-based work outside sport has shown that adenosine-related individual differences modify caffeine’s behavioral and EEG responses during sleep deprivation and recovery sleep. For sport, this supports a precision-nutrition approach: sleep EEG could serve as a mechanistic outcome to identify athletes who gain performance benefits from caffeine with minimal recovery cost versus those who show a pronounced “hyperarousal-like” EEG response and impaired nocturnal recovery. This is highly relevant for individualized caffeine protocols in competition phases.

Fourth, sleep EEG provides a stronger bridge between laboratory caffeine studies and applied sport settings than subjective sleep ratings alone. Athletes often underreport or poorly detect sleep disruption—especially when they feel acutely stimulated or perform well in the short term [104]. Similar subjective–objective dissociations have been reported in broader sleep/caffeine research, and athlete-monitoring studies also show that subjective wellness does not always track objective sleep changes [105,106]. EEG-derived metrics can therefore serve as more sensitive biomarkers in intervention studies, helping researchers detect recovery impairment before it manifests as an apparent decrement in total sleep time or next-day performance.

In practical sports-research terms, the importance of caffeine’s effects on sleep-related EEG is therefore threefold. First, they identify a plausible mechanism by which evening or repeated caffeine use may reduce the restorative value of sleep despite preserving immediate alertness. Second, they provide a biologically meaningful endpoint for individualized caffeine prescriptions (dose, timing, competition-day use, travel use). Third, they offer a way to integrate ergogenic and recovery science: rather than treating caffeine solely as a performance aid, sport research can evaluate it as a performance–recovery trade-off intervention, with sleep EEG as the mechanistic marker of recovery.

Future sport-specific studies should build on this by combining: (i) ecologically valid caffeine protocols (competition-like timing, repeated dosing, travel scenarios), (ii) high-resolution sleep outcomes (portable PSG/EEG, spectral and spindle analyses), and (iii) next-day sport-relevant endpoints (reaction time, decision-making, neuromuscular performance, technical skill retention, and injury-risk proxies). This would allow the field to move beyond “does caffeine disturb sleep?” toward the more useful applied question: when, for whom, and at what cost to overnight neural recovery and subsequent adaptation does caffeine improve performance?

## 6. Limitations and Future Directions

### 6.1. Restricted Sample Composition and Limited Generalizability

A major limitation across the studies is the narrow composition of many samples. Several experiments relied on small cohorts of healthy young. In contrast, older women, adolescents, older adults, and individuals with comorbid sleep or psychiatric vulnerability were often underrepresented or studied only in isolated protocols. This matters because caffeine pharmacokinetics and sleep responses vary with age, sex hormones, habitual use, and trait sensitivity [22,34]. For example, oral contraceptive use reduces CYP1A2-mediated caffeine clearance and prolongs caffeine half-life, which can materially change bedtime exposure even when nominal doses remain the same [107]. Genetic variability also contributes to heterogeneous sleep responses, especially at the ADORA2A locus. Future studies should therefore recruit larger, sex-balanced, age-diverse samples and explicitly stratify or adjust for hormonal status, contraceptive use, menopausal stage, genotype, and habitual caffeine intake rather than treating them as background noise.

A related issue is that “healthy moderate consumers” dominate the field, which improves internal control but weakens external validity. Real-world caffeine use is common in adolescents, shift workers, and people with irregular sleep schedules, and ambulatory data suggest that timing-sensitive effects can be substantial outside the lab, especially later in the day. Future work should include more clinically and ecologically relevant groups, including individuals with insomnia vulnerability, circadian misalignment, or high real-world caffeine exposure.

This restricted sampling has important implications for extrapolation. The central conclusion of this review—that caffeine tends to suppress low-frequency NREM activity, attenuate slow-wave activity, and shift the sleep EEG toward a lighter, more aroused profile—appears robust in tightly controlled studies of healthy adults. However, the extent to which this pattern generalizes to underrepresented populations is less clear. In women, differences in hormonal status and oral contraceptive use may alter caffeine clearance, prolonging biologically relevant exposure at sleep onset. In older adults, age-related changes in sleep architecture, homeostatic dynamics, and drug handling may amplify or qualitatively alter the observed effects. In adolescents, ongoing neurodevelopment, high baseline sleep need, and school-related circadian constraints may make caffeine-related disruption particularly relevant even when the absolute doses appear modest. Likewise, in heavy habitual users, tolerance, withdrawal dynamics, and different baseline adenosinergic adaptation may modify both the magnitude and the detectability of EEG disruption. Thus, the review’s conclusions should be interpreted as most directly applicable to healthy low-to-moderate consumers under laboratory conditions, rather than as uniformly transferable to all caffeine-consuming populations.

### 6.2. Incomplete Control of Dose, Timing, and Withdrawal State

Another limitation is imperfect exposure characterization. Even when nominal caffeine doses are standardized, the actual biological exposure at sleep onset can differ substantially because of inter-individual differences in absorption, metabolism, body mass, hormone status, and prior intake. This complicates comparisons across studies using “the same” dose. Broader evidence now shows that both dose and timing strongly shape sleep disruption: a large systematic review found consistent reductions in total sleep time and sleep efficiency with caffeine, while timing studies indicate that high doses can impair sleep even when taken 6 h before bedtime. Higher doses may remain disruptive for up to 12 h after bedtime. Future EEG studies should therefore model exposure continuously, using plasma or salivary caffeine and metabolite measures. They should report dose relative to body mass, timing relative to both bedtime and circadian phase, and time since last habitual use.

Withdrawal is another persistent confound. In habitual consumers, withdrawal symptoms can begin within 12–24 h and peak roughly 20–51 h after cessation, meaning that short abstinence windows may not create a true “caffeine-free” baseline but instead a mixed state of declining exposure and emerging withdrawal [108]. This is especially relevant for crossover studies and chronic-use protocols. Future protocols should clearly distinguish between acute administration, residual exposure, overnight abstinence, and bona fide withdrawal, ideally by adding longer washout periods, preregistered abstinence criteria, and repeated biomarker sampling throughout the protocol.

### 6.3. Measurement Heterogeneity and Comparability Across Studies

The studies included used a wide range of recording systems, derivations, staging approaches, spectral bands, and analytical pipelines. That diversity is useful for innovation, but it reduces comparability across findings. Some studies used full laboratory polysomnography, whereas others used mobile or wearable EEG systems. Wearable devices can estimate summary sleep variables reasonably well, but wake detection, awakenings, and stage classification remain less robust than gold-standard PSG, and performance varies across devices and populations. Although newer validations of the Dreem platform are encouraging, broader wearable validation work still suggests that consumer and semi-mobile systems are best interpreted cautiously for staging and microarchitecture. Future research should prioritize harmonized preprocessing, shared spectral definitions, standardized reporting of artifact rejection, and direct PSG-validation subsamples whenever mobile EEG is used.

A further measurement issue is that conventional sleep architecture and EEG microstructure do not always move together. Broader caffeine work shows that subjective sleep and standard PSG outcomes can appear only modestly changed even when timing-sensitive or microstructural disruptions are present. This argues for multimodal outcome sets that combine macrostructure, spectral, and aperiodic EEG measures, autonomic indices, next-day functioning, and subjective recovery. Future studies should also assess reproducibility across nights, because one-night effects may reflect first-night adaptation, expectancy, or stochastic state fluctuations rather than stable caffeine physiology.

### 6.4. Limited Ecological Validity of Laboratory Paradigms

Many current paradigms maximize control by using total sleep deprivation, fixed bedtimes, or tightly supervised lab routines. These designs are valuable mechanistically, but they do not fully represent everyday caffeine behavior, which is often distributed across the day, combined with social jetlag, and embedded in home environments. Ambulatory adolescent data and home-based timing studies show that caffeine’s sleep effects can depend strongly on naturalistic timing patterns and may be underestimated by self-report. Future work should combine laboratory precision with ecological validity through hybrid designs: repeated at-home EEG or PSG-quality monitoring, digital diaries, biomarker sampling, and within-person modeling of day-to-day caffeine timing.

This is especially important because individuals may not accurately perceive the degree of caffeine-related sleep disruption. Objective home monitoring studies indicate that sleep loss and fragmentation can be measurable even when subjective deterioration is modest. Future studies should therefore treat subjective and objective sleep as complementary rather than interchangeable and should explicitly test misperception as a meaningful outcome.

### 6.5. Need for Mechanistic and Precision-Medicine Approaches

The next phase of the field should move beyond asking whether caffeine changes sleep EEG to asking in whom, through which pathways, and under which circadian conditions those effects emerge. Existing pharmacogenetic evidence shows that ADORA2A variation contributes to caffeine-related sleep sensitivity, and observational genetic work suggests that these differences extend into habitual behavior and self-reported sleep vulnerability. Yet most experimental studies remain underpowered for genotype-by-treatment analyses. Future directions should include larger multi-site studies with genetic stratification, formal tests of gene-by-dose and gene-by-circadian-phase interactions, and integration of CYP1A2-related metabolism markers with receptor-level susceptibility.

Mechanistically, more work is also needed to separate homeostatic from circadian effects. Broader evidence suggests that caffeine can alter REM timing and that timing-of-day critically shapes disruption, but many EEG studies still focus mainly on NREM slow frequencies. Future studies should incorporate circadian phase markers such as dim-light melatonin onset, controlled light exposure, and scheduled sleep opportunities across different circadian windows. Combining quantitative EEG with autonomic, endocrine, pharmacokinetic, and behavioral endpoints would enable more integrated models of how caffeine perturbs sleep regulation and next-day recovery.

## 7. Conclusions

This systematic and mechanistic review shows that caffeine exerts a clear and physiologically coherent effect on sleep-related EEG in humans. Across highly diverse paradigms—including acute bedtime dosing, daytime or evening intake before nocturnal sleep, administration during prolonged wakefulness followed by recovery sleep, repeated daytime use, withdrawal designs, and ambulatory monitoring—the most consistent finding is a reduction in low-frequency NREM EEG activity, especially slow-wave activity and the lowest delta frequencies. This effect is often accompanied by relative increases in faster frequencies, including sigma and beta activity, and by sleep that appears electrophysiologically lighter, less synchronized, and in some contexts more fragmented. In many studies, these EEG changes were more sensitive than conventional sleep-stage variables, suggesting that caffeine can alter sleep physiology even when macrostructural sleep disruption appears modest.

The overall pattern strongly supports a mechanistic account centered on adenosine receptor antagonism and the attenuation of sleep homeostasis. Rather than merely promoting wakefulness at the behavioral level, caffeine appears to weaken both the build-up and the nocturnal expression of sleep pressure, particularly under conditions in which homeostatic drive should be strongest, such as early-night NREM sleep and recovery sleep after sleep deprivation. The literature also suggests that caffeine shifts brain activity during sleep toward a more aroused or wake-like state, as reflected not only in spectral power changes but also, in newer studies, in increased EEG complexity and altered dynamical organization. Although REM-related effects are less consistent than NREM effects, caffeine can still alter REM timing and REM-related EEG features under specific conditions.

At the same time, the review highlights essential moderators of effect magnitude. Dose, timing, formulation, habitual caffeine use, withdrawal state, circadian context, age, and inter-individual biological susceptibility all influence the observed EEG response. Genetic and sensitivity-related findings involving adenosinergic pathways indicate that caffeine’s sleep effects are not uniform across individuals. This variability helps explain why some people show pronounced objective sleep disruption, whereas others show weaker or more selective changes despite similar intake.

The evidence base is substantial but also limited by methodological heterogeneity, small samples in many mechanistic studies, frequent reliance on healthy young adults—often men—and the relative scarcity of high-density EEG, longitudinal designs, and clinically diverse populations. Accordingly, future research should prioritize larger and more representative cohorts, better characterization of habitual intake and metabolite exposure, integration of pharmacokinetic and pharmacogenetic measures, and more ecologically valid protocols using portable high-resolution sleep monitoring. It will also be essential to determine how caffeine-related changes in sleep EEG translate into next-day cognitive, emotional, and motor learning outcomes, as well as longer-term health and performance outcomes.

In conclusion, the human sleep-EEG literature indicates that caffeine reliably alters the neurophysiological architecture of sleep in a direction consistent with reduced sleep depth and weakened homeostatic recovery. Quantitative sleep EEG, therefore, provides a sensitive mechanistic window into caffeine’s biological effects on the sleeping brain and shows that caffeine’s primary sleep-related impact is often on sleep quality rather than sleep quantity alone. Its effects are expressed in the neurophysiological architecture of sleep, including reduced low-frequency synchronization and relatively greater, faster-frequency, wake-like activity, even when total sleep time or conventional sleep-stage measures appear only modestly changed. Thus, even when sleep duration seems broadly preserved, the brain may remain in a more activated, less restorative state during sleep. In this sense, the physiological cost of caffeine is not limited to delayed sleep onset or shorter sleep duration; it also includes a shift toward a lighter, more aroused, and more wake-like form of sleep. Even when subjective sleep quality or conventional sleep staging appears only mildly affected, the sleeping brain often carries a detectable electrophysiological signature of caffeine exposure.

## Figures and Tables

**Table 1:** Effects of caffeine on acute nocturnal sleep.

Main Moderating Finding	EEG/Sleep Outcomes Most Affected	Sleep Context	Caffeine Exposure	Population/Moderator	Experimental Design Subgroup	Study
REM effects stronger in young adults; NREM effects broadly similar across age groups	↑ EEG complexity/entropy, ↓ DFA and aperiodic slope; NREM: ↓ delta/theta/alpha, ↑ beta	Ordinary overnight sleep	200 mg evening/night	Healthy adults, 20–58 y; young vs. middle-aged subgroup	Acute nocturnal sleep	[11]
Effect size depended on plasma caffeine concentration, not just dose	Concentration-dependent ↓ NREM delta (0.75–2.5 Hz), ↓ N3; threshold around 7.3–7.4 μmol/L for delta suppression	4 h sleep opportunity with rising in-sleep caffeine	160 mg delayed-release bedtime capsule	Healthy young men	Acute nocturnal sleep	[13]
Strongest effect confined to first NREM episode; most whole-night effects attenuated next night	Early-night ↓ SWA/SWS, slower SWA build-up in first NREM episode, slight spindle-range ↑	Ordinary overnight sleep + post caffeine night	100 mg at bedtime	Healthy young men	Acute nocturnal sleep	[14]
Residual low bedtime levels still associated with nocturnal EEG changes	NREM: ↓ very-low delta, ↑ spindle activity; REM: ↓ 0.75–6 Hz	Ordinary overnight sleep	200 mg in the morning	Healthy young men	Acute nocturnal sleep	[15]
Older subgroup within sample showed larger TST loss and SOL increase	Marked sleep disruption; early-night ↓ SWS, ↑ wake/stage 1	Ordinary overnight sleep	300 mg at bedtime	Late middle-aged adults (50–63 y)	Acute nocturnal sleep	[19]
Trazodone and zolpidem partly reversed caffeine-induced sleep disruption	↓ sleep efficiency/TST, ↑ SOL; trend toward reduced first-cycle SWA and altered delta/theta ratios	Ordinary overnight sleep	150 mg at bedtime	Healthy young men	Acute nocturnal sleep/insomnia model	[20]
Standard PSG effects similar by age; only limited age-specific EEG-bin differences	↓ low-frequency power, ↑ beta/high-frequency activity; ↓ TST/SE, ↑ SOL	Ordinary overnight sleep	200 mg evening (100 mg at −3 h, 100 mg at −1 h)	Young vs. middle-aged adults	Acute nocturnal sleep	[22]
Age difference emerged mainly at 400 mg: middle-aged adults more sleep-disrupted	↓ low-frequency power, ↑ 14–19 and 27–32 Hz; ↓ TST/SE, ↑ SOL, ↓ SWS	Ordinary overnight sleep	200 vs. 400 mg evening split dose	Young vs. middle-aged adults	Acute nocturnal sleep, dose comparison	[23]
Greater SWS suppression in those with higher placebo-night SWS; DLMO shift varied by relative mg/kg dose	↓ SWS proportion; little group-level DLMO effect	Ordinary overnight sleep	80 mg evening (~4 h before bedtime)	Male adolescents	Acute nocturnal sleep/adolescent sample	[25]
Confirms dose-response; caffeine accounted for most coffee effect	Dose-related disruption; delayed sleep onset, ↓ TST/SE, altered REM/SWS distribution across night	Ordinary overnight sleep	Coffee/caffeine 1-, 2-, 4-cup equivalents; caffeine-only 4.6 mg/kg	Healthy young men	Acute nocturnal sleep, dose-response	[26]
Significant objective disruption even at 6 h before bedtime	↓ TST/SE, ↑ wake time; reduced stage 1 + 2 and SWS	Ordinary overnight sleep at home	400 mg at bedtime, −3 h, or −6 h	Healthy adults	Acute nocturnal sleep, timing study	[37]
Timing mattered mainly for 400 mg; stronger effects closer to bedtime	400 mg impaired TST/SE/SOL/WASO and ↓ N3; 100 mg no significant effect	Ordinary overnight sleep at home	100 vs. 400 mg at −12 h, −8 h, or −4 h	Healthy men	Acute nocturnal sleep, dose × timing study	[40]
High sleep-reactivity group showed much larger SOL increase	Main effect on sleep onset; trend toward reduced SWS in high-reactivity group	Ordinary overnight sleep	3 mg/kg at bedtime	Healthy sleepers with low vs. high sleep reactivity (FIRST)	Acute nocturnal sleep/vulnerability model	[39]

**Table 2:** Effects of caffeine on recovery sleep after sleep deprivation/nap paradigms.

Main Moderating Finding	EEG/Sleep Outcomes Most Affected	Sleep Context	Caffeine Exposure	Population/Moderator	Experimental Design Subgroup	Study
Supports attenuation of sleep-pressure build-up rather than simple stimulation	Waking: ↓ theta; Recovery sleep: ↓ 0.75–2 Hz, ↑ 11.25–20 Hz	Recovery night after 40 h wakefulness	200 mg twice during 40 h wakefulness	Healthy young men	Recovery sleep after total sleep deprivation	[10]
Higher habitual caffeine intake predicted poorer recovery sleep, especially with acute caffeine	↓ delta recovery power, ↓ N3, ↑ WASO, more fragmentation and stage transitions	Recovery night after TSD	2.5 mg/kg repeated during 38 h wakefulness	Healthy adults; habitual intake as covariate	Recovery sleep after total sleep deprivation	[12]
Habitual use only modestly moderated disruption	High dose: ↓ TST/SE, ↑ stage 1, delayed SWS onset; little REM effect	8 h recovery sleep after 27 h wakefulness	Repeated gum doses totaling ~255–765 mg	Healthy adults; low vs. high habitual users	Recovery sleep after prolonged wakefulness	[16]
Age affected baseline daytime sleep quality, but caffeine effect was broadly similar across ages	↓ NREM synchronization; ↑ 14–19 Hz, ↓ 4–12 Hz; ↓ SWS/REM, ↑ latency	Daytime recovery sleep after 25 h wakefulness	200 mg daytime split dose	Young vs. middle-aged adults	Daytime recovery sleep after deprivation	[17]
Circadian context magnified caffeine’s disruptive effect	Caffeine impaired both, but much stronger reduction in consolidation during daytime recovery sleep	Night sleep vs. daytime recovery sleep after deprivation	200 mg evening split dose	Healthy adults, matched Night vs. DayRec groups	Night sleep vs. daytime recovery sleep	[18]
Important “negative” study: stimulant benefit during wakefulness did not clearly impair later recovery sleep	No meaningful EEG/PSG disruption of recovery sleep overall	Two recovery nights after deprivation	Slow-release caffeine 300 mg twice daily during 64 h wakefulness	Healthy young men	Recovery after extreme sleep loss	[24]
Suggests increased physiological arousal can reduce restorative value without reducing total sleep	Same TST but ↑ stage 1, ↓ stage 4; poorer later performance despite equal sleep time	Fixed-duration 210-min sleep episode before prolonged wakefulness	400 mg before short nocturnal nap	Healthy young men	Nap/restorative sleep paradigm	[28]
Caffeine mainly reduced sleep inertia, not general vigilance across the whole protocol	Modest nap-sleep changes; targeted reduction in post-nap performance impairment	2 h naps every 12 h during sleep loss	0.3 mg/kg hourly during extended wakefulness	Healthy adult men	Repeated nap/sleep inertia paradigm	[29]
Best classified as operational fatigue-countermeasure study rather than pure sleep-EEG disruption	Main EEG-related outcome was prolonged MSLT latency (greater alertness) rather than spectral sleep effects	4 h prophylactic nap and subsequent sleep-loss period	Sustained-release caffeine 200 mg at 01:30 and 07:30	Healthy young men	Prophylactic nap + caffeine	[32]
Strong genotype-dependent caffeine effect	Caffeine suppressed SWA rebound in non-HT4 but not HT4 carriers	Recovery night after prolonged wakefulness	Caffeine 200 mg twice during wakefulness	Healthy men; ADORA2A haplotypes	Recovery sleep after deprivation/genetic moderation	[34]
Links caffeine sensitivity phenotype to EEG/topographic response	Caffeine modulated waking theta and recovery < 1 Hz topography; reversed deprivation effects more in sensitive men	Recovery night after 40 h wakefulness	Caffeine 200 mg twice during wakefulness	Caffeine-sensitive vs. insensitive men	Recovery sleep after deprivation/sensitivity phenotype	[36]
Genotype effect most evident in beta “insomnia-like” activity	↓ low-delta, ↑ alpha/sigma overall; beta increase strongest in C/C genotype	Recovery night after 40 h wakefulness	Caffeine 200 mg twice during wakefulness	Caffeine-sensitive vs. insensitive/genotype-defined	Recovery sleep after deprivation/ADORA2A genotype	[30]

**Table 3:** Habitual use/withdrawal/naturalistic exposure.

Main Moderating Finding	EEG/Sleep Outcomes Most Affected	Sleep Context	Caffeine Exposure	Population/Moderator	Experimental Design Subgroup	Study
Timing was critical; evening use produced the largest effects	More caffeine, especially evening intake, predicted ↓ TST/SE/REM and ↑ SOL	Naturalistic home sleep over 7 nights	Daily self-reported caffeine timing/amount	Adolescents	Habitual/day-to-day naturalistic use	[21]
Withdrawal effects were clearer than ongoing caffeine effects	Ongoing use: little circadian change; Withdrawal: ↑ sleepiness, longer nap TST, ↑ SWS, shorter sleep latencies	Circadian protocol + evening nap	150 mg three times/day for 10 days vs withdrawal	Habitual adult male users	Habitual use vs withdrawal	[27]
Bedtime xanthine levels did not explain most sleep differences	Little association with PSG/EEG; only modest link with stage 1	Single PSG night	Real-life caffeine use indexed by plasma caffeine + paraxanthine	Primary insomnia vs good sleepers	Habitual low–moderate use/clinical-naturalistic sample	[31]
Microstructure changed despite null standard sleep outcomes	No PSG macrostructure effects, but ↓ sigma power in both caffeine and withdrawal	Ordinary overnight PSG	150 mg three times/day for 10 days; withdrawal on day 9	Healthy habitual male users	Habitual use vs withdrawal, lab crossover	[33]
Suggests regular daytime caffeine can alter REM timing without broad NREM disruption	Delayed REM latency/REM accumulation in caffeine condition; no major SWS/delta change	Sleep scheduled at circadian REM peak	150 mg three times/day for 10 days; withdrawal	Healthy habitual male users	Habitual daytime use/circadian REM study	[35]
Shows adaptation over time but incomplete tolerance	Acute: ↓ TST/SE, ↑ SOL/Wake; later partial tolerance; persistent stage 4 reduction	Repeated nights + withdrawal	Sustained-release caffeine 400 mg three times/day for 1 week	Healthy young men	Repeated high-dose use/insomnia model	[38]

**Table 4:** Risk of bias assessment (RoB-2).

Bias in Selection of the Reported Result	Bias in Measurement of the Outcome	Bias Due to Missing Outcome Data	Bias Due to Deviations from Intended Interventions	Bias Arising from the Randomization Process	Study
Some concerns	Low risk (objective EEG); Some concerns (subjective sleepiness)	Low risk	Low risk	Some concerns	[10]
Some concerns	Some concerns	Low risk	Low risk	Some concerns	[11]
Some concerns	Low risk	Some concerns	Low risk	Some concerns	[12]
Some concerns	Low risk	Some concerns	Low risk	Some concerns	[13]
Some concerns	Low risk	High risk	Low risk	Some concerns	[16]
Some concerns	Low risk	Low risk	Low risk	Some concerns	[17]
Some concerns	Low risk	Low risk	Low risk	Some concerns	[18]
Some concerns	Low risk	Low risk	Low risk	Some concerns	[19]
Some concerns	Low risk	Low risk	Low risk	Some concerns	[20]
Some concerns	Low risk	Low risk to some concerns	Low risk	Some concerns	[22]
Some concerns	Low risk	Some concerns	Low risk	Some concerns	[23]
Some concerns	Low risk	Low risk	Low risk	Some concerns	[24]
Some concerns	Low risk	Some concerns	Low risk	Some concerns	[25]
Some concerns	Low risk	Low risk	Low risk	Some concerns	[26]
Some concerns	Low risk	Some concerns	Low risk	Some concerns	[27]
Some concerns	Low risk for objective performance outcomes; Some concerns for subjective outcomes	Low risk	Some concerns	Some concerns	[28]
Some concerns	Low risk	Low risk	Low risk	Some concerns	[29]
Some concerns	Low risk	Some concerns	Low risk	Some concerns	[30]
Some concerns	Low risk to some concerns	Low risk	Some concerns	Some concerns	[32]
Some concerns	Low risk	Some concerns	Low risk	Some concerns	[33]
Some concerns	Low risk	Low risk	Low risk	Some concerns	[34]
Some concerns	Low risk	Some concerns	Low risk	Some concerns	[35]
Some concerns	Low risk	Some concerns	Low risk	Some concerns	[36]
Some concerns	Low risk for objective TST; Some concerns for self-reported sleep diary outcomes	Some concerns	Low risk	Some concerns	[37]
Some concerns	Low risk for objective sleep outcomes; Some concerns for subjective outcomes	Some concerns	Some concerns	Low risk	[40]
Some concerns	Low risk for objective sleep outcomes (EEG sleep, MSLT); Some concerns for subjective outcomes (LSEQ, LARS)	Low risk	Some concerns	Some concerns	[41]

**Table 5:** Risk of bias assessment (ROBINS-I).

Bias in Selection of the Reported Result	Bias in Measurement of Outcomes	Bias Due to Missing Data	Bias Due to Deviations from Intended Interventions	Bias in Classification of Interventions	Bias in Selection of Participants into the Study	Bias Due to Confounding	Study
Moderate	Moderate	Low	Low to Moderate	Low	Low	Serious	[14]
Serious	Low to Moderate	Low	Moderate	Low	Low	Serious	[15]
Moderate to Serious	Moderate	Moderate	Low	Serious	Moderate	Serious	[21]
Moderate	Low	Moderate	Low	Moderate	Moderate	Serious	[31]
Moderate	Moderate	Low	Moderate to Serious	Low	Moderate	Serious	[38]
Moderate	Low to Moderate	Moderate	Serious	Low	Moderate	Serious	[39]

## Data Availability

No new data were created or analyzed in this study. Data sharing does not apply to this article.
